# Just-in-Time Adaptive Intervention for Stabilizing Sleep Hours of Japanese Workers: Microrandomized Trial

**DOI:** 10.2196/49669

**Published:** 2024-06-11

**Authors:** Hiroki Takeuchi, Tetsuro Ishizawa, Akifumi Kishi, Toru Nakamura, Kazuhiro Yoshiuchi, Yoshiharu Yamamoto

**Affiliations:** 1 Graduate School of Education The University of Tokyo Tokyo Japan; 2 Graduate School of Medicine The University of Tokyo Tokyo Japan; 3 Central Medical Support Co Tokyo Japan; 4 Institute for Datability Science Osaka University Osaka Japan

**Keywords:** objective push-type sleep feedback, stability of habitual sleep behaviors, just-in-time adaptive intervention, microrandomized trial, mobile phone

## Abstract

**Background:**

Sleep disturbance is a major contributor to future health and occupational issues. Mobile health can provide interventions that address adverse health behaviors for individuals in a vulnerable health state in real-world settings (just-in-time adaptive intervention).

**Objective:**

This study aims to identify a subpopulation with vulnerable sleep state in daily life (study 1) and, immediately afterward, to test whether providing mobile health intervention improved habitual sleep behaviors and psychological wellness in real-world settings by conducting a microrandomized trial (study 2).

**Methods:**

Japanese workers (n=182) were instructed to collect data on their habitual sleep behaviors and momentary symptoms (including depressive mood, anxiety, and subjective sleep quality) using digital devices in a real-world setting. In study 1, we calculated intraindividual mean and variability of sleep hours, midpoint of sleep, and sleep efficiency to characterize their habitual sleep behaviors. In study 2, we designed and conducted a sleep just-in-time adaptive intervention, which delivered objective push-type sleep feedback messages to improve their sleep hours for a subset of participants in study 1 (n=81). The feedback messages were generated based on their sleep data measured on previous nights and were randomly sent to participants with a 50% chance for each day (microrandomization).

**Results:**

In study 1, we applied hierarchical clustering to dichotomize the population into 2 clusters (group A and group B) and found that group B was characterized by unstable habitual sleep behaviors (large intraindividual variabilities). In addition, linear mixed-effect models showed that the interindividual variability of sleep hours was significantly associated with depressive mood (β=3.83; *P*=.004), anxiety (β=5.70; *P*=.03), and subjective sleep quality (β=−3.37; *P*=.03). In study 2, we found that providing sleep feedback prolonged subsequent sleep hours (increasing up to 40 min; *P*=.01), and this effect lasted for up to 7 days. Overall, the stability of sleep hours in study 2 was significantly improved among participants in group B compared with the participants in study 1 (*P*=.001).

**Conclusions:**

This is the first study to demonstrate that providing sleep feedback can benefit the modification of habitual sleep behaviors in a microrandomized trial. The findings of this study encourage the use of digitalized health intervention that uses real-time health monitoring and personalized feedback.

## Introduction

### Importance and Necessity of Addressing Vulnerable Sleep States

Sleep plays a key role in preventing future adverse health outcomes, such as coronary heart disease [1,2], type 2 diabetes [2-4], and Alzheimer disease [5,6]. In addition to being relevant to chronic diseases, sleep disturbance is associated with impaired psychological wellness, including psychiatric disorders [7,8] and disruption of daytime moods [9,10], and affects work productivity (presenteeism) [11-13] and cognitive function [14,15]. In 2015, the National Sleep Foundation formulated age-specific sleep duration recommendations to prevent future health problems and recommended 7 to 9 hours of sleep for young adults and adults (aged 18-64 years) [16]. However, a recent meta-analysis of 36 epidemiological studies revealed that approximately 1 in 4 people (24.5%) slept less than the recommended amount for their age groups [17]. This suggests the difficulty of self-management for habitual sleep behaviors and the necessity for support in daily life.

### Mobile Health

Mobile health (mHealth) [18,19] is a medical health practice that uses digital devices, including smartphone apps and wearable devices. Major mHealth methodologies include collecting health-related data in real-world settings (real-world data), conducting interventions for adverse health behaviors using digital devices, and generating evidence from the collected real-world data (real-world evidence). Just-in-time adaptive intervention (JITAI) is an appealing mHealth application that supports the modification of health behaviors for individuals in a vulnerable health state [20-22]. JITAI can alleviate current health problems and prevent future disease onset by making individuals aware of their daily health behaviors through timely and adaptive interventions.

### Recent Needs for Real-World Evidence

Recently, the methodology of mHealth has received increased attention, even in the general health care field, with growing interest in leveraging real-world data. This has been triggered by the establishment of the 21st Century Cures Act in the United States and the Food and Drug Administration’s recommendation for using real-world evidence as well as evidence from gold standard clinical trials (randomized controlled trials) [23,24]. Real-world data are expected to become an essential source for monitoring an individual’s health status outside the clinic and evaluating the effectiveness of clinical and health trials. However, this trend calls for novel approaches to generate real-world evidence for the effectiveness of treatments conducted in real-world settings [25]. Microrandomization, or microrandomized trial (MRT), is a trial design used to generate real-world evidence for intervention options in mHealth trials, including JITAI [26,27]. In MRT, a specific intervention option is randomly provided for each individual via their digital device, allowing for the examination of the within-individual proximal effect by comparing subsequent behaviors with and without the intervention. For instance, Klasnja et al [28] randomly provided healthy adults with contextually tailored suggestions that encouraged physical activity during a 6-week trial period. By comparing the subsequent physical activity with and without suggestions, they revealed that providing suggestion messages proximally increased subsequent step counts and that this effect attenuated by day.

### mHealth Sleep Intervention

Advancements in mobile and wearable sensing technologies enable the objective measurement of habitual sleep behaviors across multiple days [29-31]. Thus, night-to-night sleep dynamics (eg, stability and transition in sleep data) can also be captured in a real-world setting. Despite the rapid growth in the use of real-world sleep data, a prior systematic review indicated that there are few mHealth trials leveraging objective sleep data recorded by wearable devices [32]. Hence, incorporating wearable-based sleep monitoring into mHealth trials for controlling habitual sleep behaviors is challenging. Furthermore, using push-type feedback based on the objective sleep monitoring may promote the improvement of habitual sleep behaviors. For example, according to the control theory on health behavior change [33], which has been applied to numerous health intervention trials [34-36], individuals monitor their current health state and change their behavior to reduce the discrepancy against the ideal state or set point. However, individuals may not be able to accurately recognize their current sleep state in daily lives without external feedback. Therefore, providing objective push-type sleep feedback may assist self-regulation of habitual sleep behaviors through enhancing the awareness for their current sleep state. Recently, we conducted a formative mHealth trial that provided push-type feedback messages based on objective sleep monitoring for Japanese workers [37]. The stabilized habitual sleep behavior and improved physical symptoms upon waking up were observed in the workers who received sleep feedback every day, suggesting the utility of wearable-based sleep monitoring and objective push-type sleep feedback. As the next step, we aimed to achieve sleep JITAI by identifying individuals in a vulnerable sleep state and designing appropriate intervention options to alleviate their sleep problems. Moreover, by implementing an MRT, it should be examined whether individuals immediately change their sleep behavior in response to the sleep feedback (proximal effect).

### Aim of This Paper

In study 1, we aimed to monitor the sleep of Japanese workers to identify a subpopulation that had problems in their habitual sleep behaviors. Considering the close link between sleep and psychological wellness, we measured momentary symptoms using a smartphone-based ecological momentary assessment (EMA) during the study. In study 2, we aimed to conduct an MRT to test whether providing sleep feedback messages improved subsequent sleep behaviors and psychological wellness proximally in real-world settings. In addition to the within-individual proximal effects, we aimed to examine the distal effects of the sleep JITAI using a pretest-posttest comparison design, which may provide a comprehensive understanding of the effectiveness of this trial.

## Methods

### Study 1

#### Study Design

Using a field data sampling method, we conducted an observational study of Japanese workers to reveal the sleep demographics of the current Japanese society and identify a subpopulation that needed support to improve their habitual sleep behaviors. Habitual sleep behaviors were objectively measured using a wearable device. Momentary symptoms were recorded on a smartphone app in real time to examine the association between psychological wellness and habitual sleep behavior with ecological validity. Baseline data, including demographic characteristics, pretrial psychological symptoms, and chronotype, were also measured before the survey.

#### Health Care Internet of Things System

We developed a cloud-based health care Internet of Things (HIT) system that can continuously acquire health-related information, including momentary symptoms, biological signals, and surrounding environmental information, that are recorded as parts of daily living [37,38]. The HIT system consists of a cloud server and a smartphone app for data collection (HIT server and HIT app, respectively). The HIT app is equipped with an EMA, and users can record momentary symptoms in daily life. In addition, the HIT app can connect with various digital devices, including the proprietary activity monitor used in this study (refer to the Activity Monitor section), using Bluetooth Low Energy (BLE). The data were transferred from the devices to the HIT server. The HIT server can store, integrate, and manage data uploaded from the app and send personalized messages (push-type feedback messages) to HIT app users (Multimedia Appendix 1).

#### Participants

Recruitment was conducted from January 17 to January 31, 2022. Author TI sent digital flyers to health managers of the contracted companies. Subsequently, these health managers forwarded the flyers to their employees. Workers who agreed to participate in the study were asked to access and complete the web-based registration form via QR code provided on the flyers. Inclusion criteria involved participants that are Japanese speakers, aged >18 years, and have a personal smartphone. Workers who had difficulties or dangers while using their smartphones during the day, such as above-ground workers and long-distance drivers, were excluded.

As we needed to identify individuals in a vulnerable sleep state and commence study 2 immediately after completing study 1, it was necessary to minimize variations in survey dates across participants. Hence, the sample size for study 1 was set to the number of workers who responded to the web-based registration form within the recruitment period.

#### Baseline Questionnaire

Before the survey, the participants completed a baseline questionnaire to assess their demographic characteristics, psychological symptoms, and chronotype. The items included in the questionnaire are listed in the following sections.

##### Demographic Characteristics

Participants were asked to indicate their age, sex, BMI, clinical history, and employment status (full-time employee, contingent worker, part-time worker, or other) as their demographic information. On the basis of the occupational classification table published by the Ministry of Health, Labour, and Welfare in Japan [39], they were asked to select their occupation from 12 categories: managerial, technical, sales, service, security, agricultural, productive, transportation, machinery, construction, cleaning, and others.

##### Psychological Symptoms

The pretrial psychological symptoms included the Japanese version of the Beck Depression Inventory second edition (BDI-II) [40], the State-Trait Anxiety Inventory-JZY (STAI) [41], and the Positive and Negative Affect Schedule (PANAS) [42,43]. The BDI-II is a 21-item self-report inventory used to measure the presence and severity of depression (score range 0-63). The BDI-II classifies individuals into 4 categories based on their overall score: minimal or no depression, 0 to 13; mild depression, 14 to 19; moderate depression, 20 to 28; and severe depression, 29 to 63. A score of ≥14 points was used as the clinical cutoff point for depression. The 20-item STAI is a standardized self-report inventory for measuring state and trait anxiety. The state anxiety subscale measures the intensity of the anxiety felt by an individual in the present, whereas the trait anxiety subscale measures how often an individual feels anxious. Scores range from 20 to 80 for each subscale, with higher scores indicating higher anxiety levels. The PANAS is a self-report questionnaire that assesses emotional well-being and measures positive and negative affect with 8 items. Higher scores indicate that individuals show positive and negative affect in daily life. In the Japanese version, the Cronbach α were 0.83 and 0.82 for positive and negative affect, respectively [43].

##### Chronotype

Chronotype, which represents an individual’s preferred timing for engaging in various daytime activities, was measured using the Japanese versions of the Morningness-Eveningness Questionnaire (MEQ) [44,45] and the Munich Chronotype Questionnaire (MCTQ) [46,47]. The MEQ consists of 19 items about morning or nighttime activities and classifies individuals into the morning, intermediate, and evening types based on the total score (score range 16-86). A higher score indicates that the individual prefers to engage in various activities in the morning (morning type). The MCTQ assumes that an individual’s sleep timing on work-free days adheres to their endogenous circadian rhythm and accumulated sleep loss rather than their social schedule. Hence, this scale calculates the midpoint between sleep onset and offset on work-free days (midsleep point on free days). Subsequently, sleep-corrected midpoint of sleep on work-free days (MSFsc) was introduced to assess chronotype after correcting the effect of sleep loss accumulated during workdays from midsleep point on free days. The MEQ score and MSFsc have been used as proxies for individual circadian phases because they are associated with physiological biomarkers of the human endogenous circadian rhythm [47,48]. In addition to MSFsc, the MCTQ can be used to calculate the discrepancy between an individual’s circadian phase and actual sleep timing (social jetlag). The absolute value (absolute social jetlag) is often used in chronobiological and sleep studies to measure this discrepancy.

#### EMA Questionnaire

EMA is a method for recording participants’ behavior, psychological state, and physical symptoms in real time and at multiple time points, allowing the collection of self-report and objective data with reliability and ecological validity [49,50]. Using the HIT app, participants answered an EMA questionnaire 5 times daily to assess momentary symptom data (eg, depressive mood, anxiety, and subjective sleep quality) in real time. Depressive mood and anxiety were scored using the Depression and Anxiety Mood Scale [51]. This scale comprises the following 9 adjectives representing mood states: “vigorous,” “gloomy,” “concerned,” “happy,” “unpleasant,” “anxious,” “cheerful,” “depressed,” and “worried.” On the basis of these 9 items, anxious (the average of “concerned,” “anxious,” and “worried” scores); positive (the average of “vigorous,” “happy,” and “cheerful” scores); and negative (the average of “gloomy,” “unpleasant,” and “depressed” scores) moods were calculated. Depressive mood scores were obtained by combining the last 2 mood scores as follows: score = (100 − [positive mood score + negative mood score])/2. In addition, participants were asked to rate their subjective sleep quality when they woke up: “How was your sleep quality last night?” These measurements were rated using a visual analog scale from 0 to 100 displayed on the screen. All scores were transferred to the HIT server immediately after completing each EMA questionnaire.

#### Activity Monitor

We used a wristband activity monitor (Sciencenet device; Sciencenet Inc) to measure participants’ habitual sleep behaviors. This device is equipped with triaxial piezoelectric accelerometers that measure 1-minute zero-crossing count data, which counts the number of times per epoch where the acceleration signal level crosses 0. The Cole-Kripke algorithm [52] with the rescoring rules by Webster et al [53] was applied to the data to identify whether the 1-minute epoch was sleep or wake. Subsequently, we estimated a square wave that maximized the coefficient of determination with the Cole-Kripke identifications [37]. The length and midpoint of the interval were defined as the sleep hours and midpoint of sleep of the day, respectively. Furthermore, sleep efficiency was evaluated by dividing the sleep hours by the duration that was classified as sleep. Details of this procedure can be found in our previous work [37]. We confirmed that the device performed at a level equivalent to the research-grade actigraphy (Ambulatory Monitors, Inc), which is widely used in clinical settings. Results of the comparative analysis are presented in Multimedia Appendix 2.

#### Study Protocol

We mailed the activity monitor, survey progression guide, informed consent form, and consent withdrawal form to the workers who responded to the web-based registration form. The BDI-II and STAI were also sent to the participants because they should not be replicated. The use of the device and the HIT app was explained in a tutorial video. Participants were instructed to complete other questionnaires on the web, whereas the BDI-II and STAI were answered on paper. After completing the informed consent form, they commenced the observational study.

During the 14-day study period, we instructed them to complete EMA questionnaires at randomly selected times within −10 minutes to +10 minutes of predetermined times (11 AM, 3 PM, and 7 PM) by sending a reminder message on their HIT app. In addition, they were asked to voluntarily complete the EMA when they woke up and went to bed. Furthermore, they were instructed to wear a wristband activity monitor on their nondominant wrist to measure their habitual sleep behaviors, except while bathing, showering, performing rigorous exercises, or engaging in any other activities likely to damage the device.

After the study, participants returned the survey instruments and written informed consent form to the University of Tokyo and received remuneration and a personalized sleep report. This sleep report included summarized information about their sleep (including sleep hours, midpoint of sleep, and sleep efficiency) and the sleep hygiene guidelines proposed by the Ministry of Health, Labour and Welfare in Japan [54]. The survey dates varied among participants, and data collection was completed on February 24, 2022.

#### Statistical Analysis

We conducted complete case analysis after excluding the data of the participants with <4 days of sleep events recorded. All analyses were performed using R (version 4.0.2; **R** Foundation for Statistical Computing). Statistical significance was defined as *P*<.05. We evaluated the intraindividual mean (IIM) and intraindividual variability (IIV) of measured sleep hours, midpoint of sleep, and sleep efficiency (IIM^(SH)^, IIM^(MID)^, IIM^(SE)^, IIV^(SH)^, IIV^(MID)^, and IIV^(SE)^, respectively). IIM and IIV were quantified by calculating the average and SD for each individual. Circular mean and SD were calculated for each individual using the “circular” package when evaluating IIM^(MID)^ and IIV^(MID)^. We adopted a hierarchical clustering algorithm (the Ward method in 6-dimensional Euclidean space) to the intraindividual statistics using the “hclust” function after standardizing these intraindividual statistics. We dichotomized the population based on the dendrogram (group A and group B), postulating that the overall sample comprised individuals in healthy and vulnerable sleep state. Then, we performed 2-tailed Welch *t* tests for intraindividual statistics between the groups to capture their characteristics of habitual sleep behaviors. When analyzing the IIV statistics, they were log transformed to satisfy the normality assumption. The same analysis was performed on the MEQ score, MSFsc, and absolute social jetlag as additional variables representing their sleep-wake schedules. Furthermore, the effect sizes (Hedges *g*) were calculated using the “effsize” package. We performed linear mixed-effect models for EMA-recorded depressive mood, anxiety, and subjective sleep quality using the “lmerTest” package to examine which aspects of habitual sleep behaviors were associated with momentary symptoms. The 95% CIs of the parameters were estimated by using the “confint” function.

### Study 2

#### Study Design

As shown in the Study 1 section we classified the workers into 2 clusters (groups A and B) and found that group B was characterized by unstable habitual sleep behaviors. On the basis of the results, in study 2, we designed and conducted a sleep JITAI for stabilizing their habitual sleep hours. Similar to study 1, habitual sleep behaviors were objectively measured using a wearable device, and momentary symptoms were assessed using a smartphone app in real time. On the basis of the measured sleep data, we sent objective push-type sleep feedback to the smartphone app with a 50% chance, enabling us to compare the subsequent sleep behaviors with and without the feedback (to examine the within-individual and proximal effects). Subjective sleep quality and emotional well-being were assessed using baseline and follow-up questionnaires to examine the whole effect (distal effect) of the trial. In particular, we tested whether the effect of the sleep JITAI was more pronounced for individuals in vulnerable state (group B) than those in healthy state (group A).

#### Participants

After completing study 1, we invited a subset of the participants in study 1 to study 2. Due to budgetary constraints, the maximum sample size of study 2 was set to 100. Therefore, we asked 67 workers in group B and 33 workers in group A to participate in the sleep JITAI. The workers in group A were randomly selected by using the “sample” function in R (version 4.0.2) regardless of age and sex. Recruitment was conducted from March 11 to March 14, 2022, by sending digital flyers with a QR code that links to a web-based registration form. As in study 1, those who agreed to participate were asked to complete the web-based form.

#### Baseline and Follow-Up Questionnaire

Only the possible outcomes for the sleep JITAI were assessed because their demographic characteristics were already assessed in study 1. The Japanese versions of the Pittsburgh Sleep Quality Index (PSQI) [55,56] and the PANAS were assessed before and after the survey. The PSQI is a self-report inventory used to assess sleep quality in the preceding month. The PSQI consists of 19 items on self-reported sleep quality, sleep latency, sleep duration, habitual sleep efficiency, sleep disturbances, use of sleeping medication, and daytime dysfunction. Scores range from 0 to 21, with a higher total score indicating poorer sleep quality. The strong reliability and validity of this questionnaire have been confirmed in a recent meta-analysis [57]. The explanation for the PANAS is described in the Psychological Symptoms section.

#### Intervention Protocol

Most protocols were similar to those used in study 1. We mailed the activity monitor, survey progression guide, informed consent form, and consent withdrawal form to the workers who responded to the web-based registration form. The participants were asked to complete the PSQI and PANAS as baseline questionnaires on the web. The participants commenced the survey after completing the baseline questionnaire and informed consent form.

During the 14-day survey period, participants were asked to complete the EMA questionnaires 5 times daily and wear a wristband activity monitor on their nondominant wrist, except while bathing, showering, performing rigorous exercises, or engaging in any other activities likely to damage the device. In this survey, at noon, the physical activity data uploaded to the HIT server were collected and analyzed to estimate the sleep hours for each individual using a local data analysis server. On the basis of the estimated sleep data, relative sleep sufficiency was calculated (difference between the IIM^(SH)^ in study 1 and estimated sleep hour), and personalized sleep feedback messages were generated and sent to the participants with a probability of 0.50 each day of the 14-day study; hence, the total number of messages that a participant could have received ranged between 0 to 14, theoretically. The messages informed the participants about their relative sleep sufficiency and requested them to plan and adjust their daytime activities, with reference to the sleep hygiene guidelines provided in study 1. The message reads: “You slept XX minutes longer (shorter) than your average yesterday. Stabilize your sleep habits with reference to sleep hygiene guidelines.” We designed the feedback sentences to adaptively change the meanings depending on the substituted sleep hours. We expected this message to act as negative feedback when sleep hours were relatively long and as an alert when sleep hours were relatively short. If sufficient physical activity data (<720 records/day) had not been uploaded to the server by noon, an alternative message was sent to the participant requesting them to confirm the BLE pairing of the activity monitor with their app: “It seems that your data have not been uploaded successfully. We will analyze the data again at 2 PM. Please check the BLE connection of your HIT app before then.” The same computation process was executed at 2 PM for the relevant participants. These processes were executed by local servers and were fully automated.

After the survey, the participants returned the survey instruments and written informed consent form to the University of Tokyo and received the personalized sleep report as in study 1. Finally, participants were requested to answer a web-based follow-up questionnaire, including the PSQI and PANAS. The survey dates varied among participants, and data collection was completed on May 18, 2022.

#### Statistical Analysis

##### Overview

We used linear mixed-effect models and hierarchical Bayesian models to exploratorily examine the within-individual proximal effects of the intervention. Mixed-effect ANOVA models were also performed to evaluate the distal effect. We conducted complete case analysis after excluding the data of the participants with <4 days of sleep events recorded. All analyses were performed using R (version 4.0.2; **R** Foundation for Statistical Computing). Statistical significance was defined as *P*<.05 or when the 95% credible interval did not include the null value.

##### Liner Mixed-Effect Model

We used the linear mixed-effect model to examine the within-individual proximal effect of the feedback message on subsequent sleep hours using the “lmerTest” package. The 95% CIs of the parameters were estimated by using the “confint” function. With reference to a model proposed in a previous study [58], this model incorporated an indicator of whether feedback was provided or not (feedback), an index representing the elapsed day from the beginning of the survey (day), and their interaction effect (feedback×day). Control variables, including sleep hours before providing feedback, age, and sex, were added to the model. Similar models were used for each group as a subgroup analysis to consider group heterogeneity in the effectiveness of the feedback. Furthermore, we stratified the feedback messages based on whether they indicated that the individual slept longer or shorter than the average for study 1 and tested whether the effects of the feedback differed depending on its content using a similar model. To verify the statistical power of the effect of the feedback, we performed ex-post power analysis in Multimedia Appendix 3.

##### Hierarchical Bayesian Model

Using hierarchical Bayesian modeling, we performed counterfactual simulations to examine (1) the number of days the feedback message was effective for subsequent sleep and (2) whether the changes in sleep hours were linked to improvements in psychological wellness (depressive mood, anxiety, and subjective sleep quality). In particular, we estimated the cascading effect of feedback on momentary symptoms in the next morning through changed sleep hours. Detailed model equations are provided in Multimedia Appendix 4. The model parameters and their 95% credible intervals were computed using the “rstan” package. The number of chains was set to 4 when performing the Markov chain Monte Carlo sampling. All iterations and burn-in samples were set to 8000 and 4000, respectively. Convergence across the 4 chains was defined as the point at which the value of the Gelman-Rubin statistic (Rhat) [59] for all parameters was <1.10. The model was run after controlling for age and sex.

##### Mixed-Effect ANOVA Model

Individual sleep statistics (IIM and IIV) were analyzed using 2 studies (studies 1 and 2)×2 groups (groups A and B) mixed-effect ANOVA to examine the distal effect of the intervention. When analyzing the IIV statistics, they were log transformed. The PSQI and PANAS scores, before and after the survey, were also analyzed using 2 time points (before and after)×2 groups (groups A and B) mixed-effect ANOVA. These ANOVA models were run after controlling for age and sex using the “lmerTest” package. The “lsmeans” package was used to perform the pairwise comparison tests with Tukey correction.

### Ethical Considerations

This nested study, including studies 1 and 2, underwent an organized single ethics review.

#### Ethics Approval

The Ethics Committee of the University of Tokyo approved these studies and the informed consent forms (approval number 21-353).

#### Informed Consent

Participants were explained the aim and procedure of this study through the mailed survey progression guide. If participants had any questions on the studies, they could contact author HT via email. When declining participation, they were asked to return the written consent withdrawal form. In addition, they could withdraw their consent by emailing a scanned consent withdrawal form to author HT.

#### Privacy and Confidentiality

When analyzing the data, an ID was assigned for each participant, and personal information was excluded. The correspondence table between individuals and IDs is stored by author HT, and the other authors could not access participants’ personal information.

#### Remuneration

In study 1, all participants who did not withdraw their consent were paid with vouchers worth at least ¥5000 (US $1=¥116). The renumeration amount was increased depending on the response rate for their EMA questionnaires. Participants whose response rate was ≥80% received vouchers worth ¥10,000.

In study 2, all participants were paid with a voucher worth ¥10,000 when starting the survey. Participants were able to withdraw their consent even after receiving remuneration.

## Results

### Study 1

#### Participant Sampling

**Figure 1:**
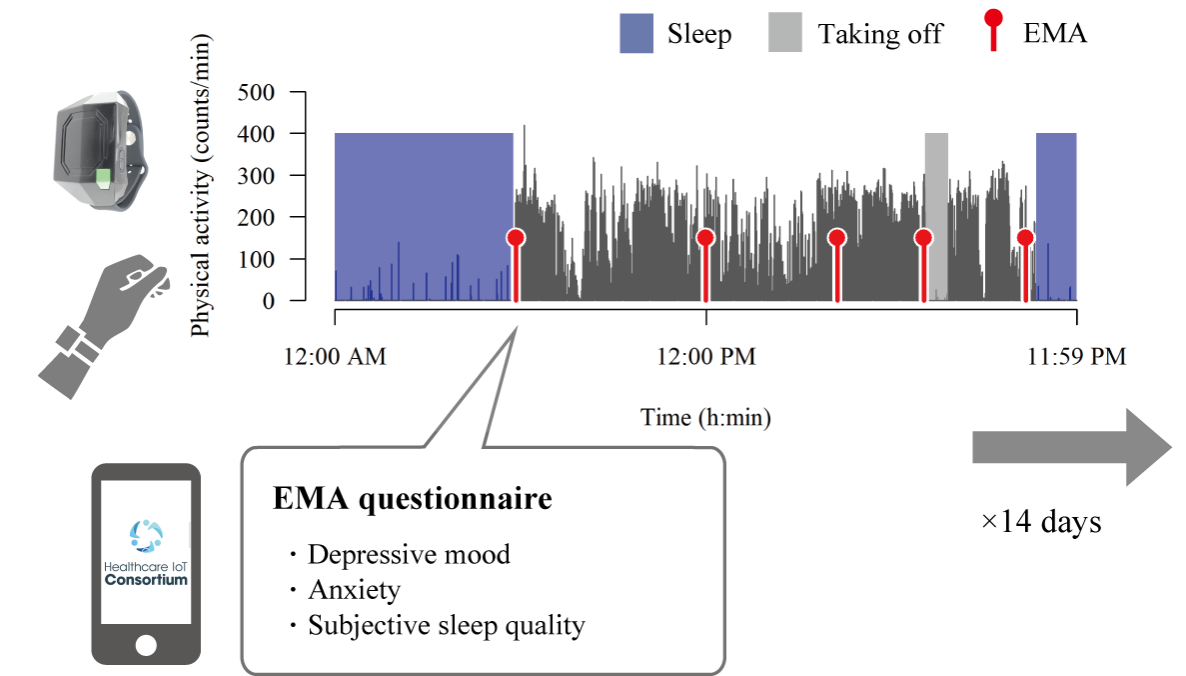
Overview of study 1. During the 14-day study period, participants wore a wristband activity monitor on their nondominant wrist to measure their sleep data and recorded momentary symptoms using smartphone-based ecological momentary assessment (EMA). Depressive mood and anxiety were assessed 5 times daily, and subjective sleep quality was assessed only when they woke up. "Taking off" refers to the period that the individual took off the device.

In total, 202 workers completed the web-based registration form, but 20 (10%) withdrew from participation before writing the consent form because of problems in installing the HIT app and unexpected work schedules (Figure S1 in Multimedia Appendix 5). Hence, 182 (90%) participants started the survey to measure their habitual sleep data and record momentary symptoms in daily life (Figure 1). Among them, 4 (2%) dropped out during the survey because of discomfort with the survey instruments. A total of 16 (9%) participants who did not return their consent forms were excluded from the analysis. Furthermore, 22 (12%) participants were excluded, as they failed to measure the sleep data, with <4 days of sleep events recorded. Finally, data from 140 (77%) participants were used for subsequent analysis. The mean age of the participants was 39.15 (SD 10.09) years, and 27.7% (29/140) were women. Table S1 in Multimedia Appendix 6 presents other baseline data and sleep variables recorded by the wearable devices.

#### Data-Driven Approach for Identifying Sleep Problems

**Figure 2:**
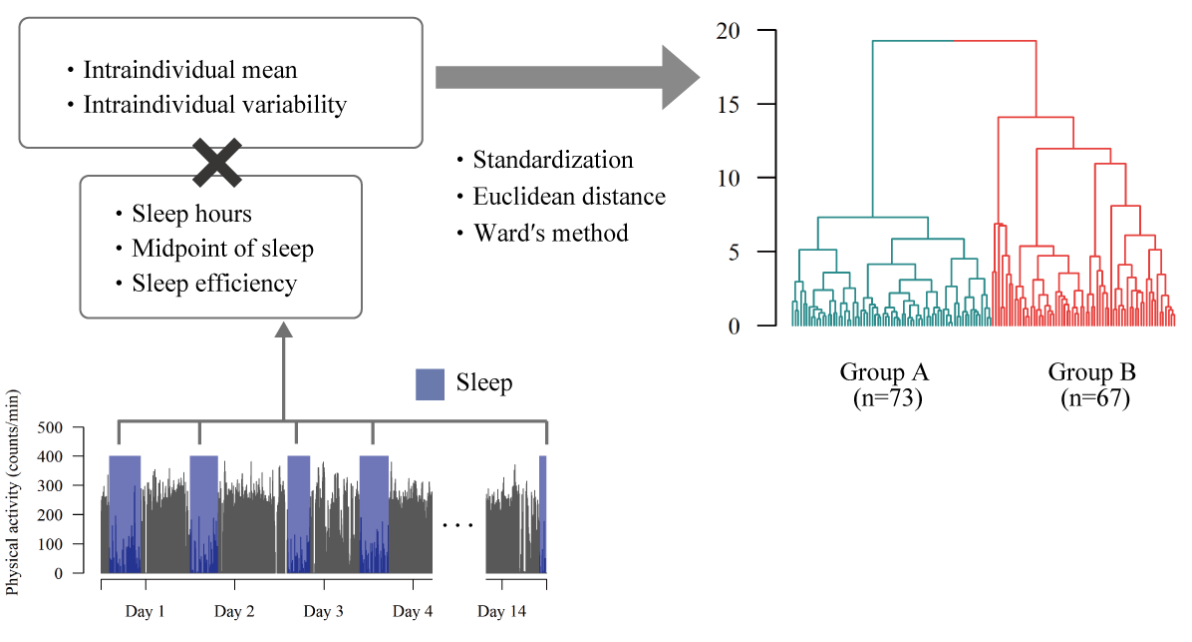
Summary of feature extraction and clustering procedure. Over the study period, objective sleep data (sleep hour, midpoint of sleep, and sleep efficiency) were collected repeatedly (up to 14 nights/person). Participants’ habitual sleep behaviors were characterized by evaluating the intraindividual mean and intraindividual variability of the measurements. After standardizing them, we performed hierarchical clustering algorithm with Ward’s method in Euclidean space to identify a subpopulation with sleep problems in daily life.

**Figure 3:**
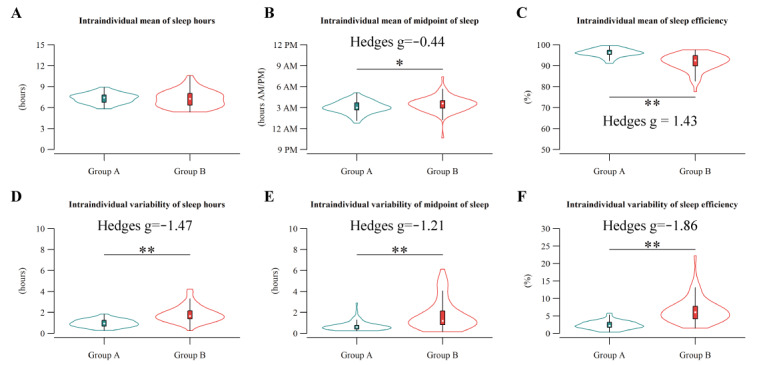
Group comparisons of intraindividual sleep statistics. Group comparison of the intraindividual mean (A-C) and intraindividual variability of sleep hours, midpoint of sleep, and sleep efficiency (D-F). Hedges g was calculated when significant group difference (*P*<.05) was observed. *P<.05; **P<.01.

On the basis of the measured sleep data (1347 nights in total), we calculated the intraindividual statistics characterizing their habitual sleep behaviors (ie, (IIM^(SH)^, IIM^(MID)^, IIM^(SE)^, IIV^(SH)^, IIV^(MID)^, and IIV^(SE)^). Subsequently, we adopted a hierarchical clustering algorithm and divided the participants into 2 clusters (Figure 2). One group (group A) consisted of 73 participants, including 55 (75%) men and 18 (25%) women, with a mean age of 39.26 (SD 9.56) years. The other group (group B) consisted of 67 participants, including 56 (84%) men and 11 (16%) women, with a mean age of 39.03 (SD 10.71) years. When performing 2-tailed Welch *t* tests, the participants in group B showed not only delayed IIM^(MID)^ (*P*=.01) and lower IIM^(SE)^ (*P*<.001) but also higher IIV^(SH)^ (*P*<.001), IIV^(MID)^ (*P*<.001), and IIV^(SE)^ (*P*<.001) than those in group A (Figure 3 and Table S2 in Multimedia Appendix 6). Furthermore, large effect sizes (Hedges *g* >0.8) were observed in the differences in IIM^(SE)^ and all the IIV statistics, implying that the participants in group B were characterized especially by the IIV aspects of their sleep data (sleep instability) rather than their IIM aspects. In addition, MEQ (*P*<.001), MSFsc (*P*<.001), and absolute social jetlag (*P*=.004) also significantly differed between the groups, with moderate effect sizes (Hedges *g*=|0.51-0.65|; Table S2 in Multimedia Appendix 6). These results suggest the necessity of an mHealth intervention to stabilize habitual sleep behaviors especially for participants in group B.

#### Associations Between IIV and Momentary Symptoms

**Figure 4:**
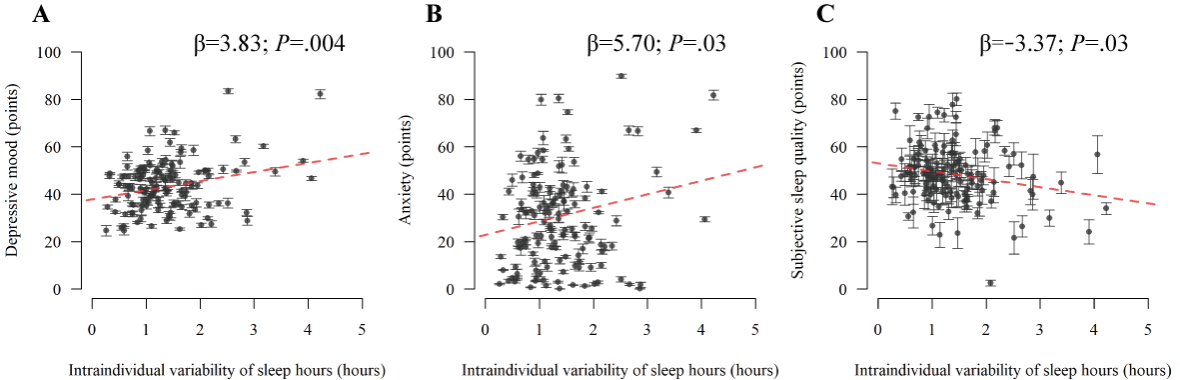
Associations between intraindividual variability of sleep hours and momentary symptoms. (A: depressive mood; B: anxiety; and C: subjective sleep quality) using linear mixed-effect models. The points and error bars represent the individual means and SEs of the momentary symptoms, respectively. The red dotted lines in the figure indicate the regression lines of intraindividual variability of sleep hours.

The EMA procedures yielded 9349 records on depressed mood and anxiety, as well as 1896 records on subjective sleep quality. We performed linear mixed-effect models to identify the aspects of IIV associated with these momentary symptoms. The models indicated a significant association between IIV^(SH)^ and these symptoms (depressive mood, *P*=.004; anxiety, *P*=.03; subjective sleep quality, *P*=.03; Figure 4 and Table S3 in Multimedia Appendix 6), implying that improving IIV^(SH)^ might benefit psychological wellness.

### Study 2

#### Participant Sampling

**Figure 5:**
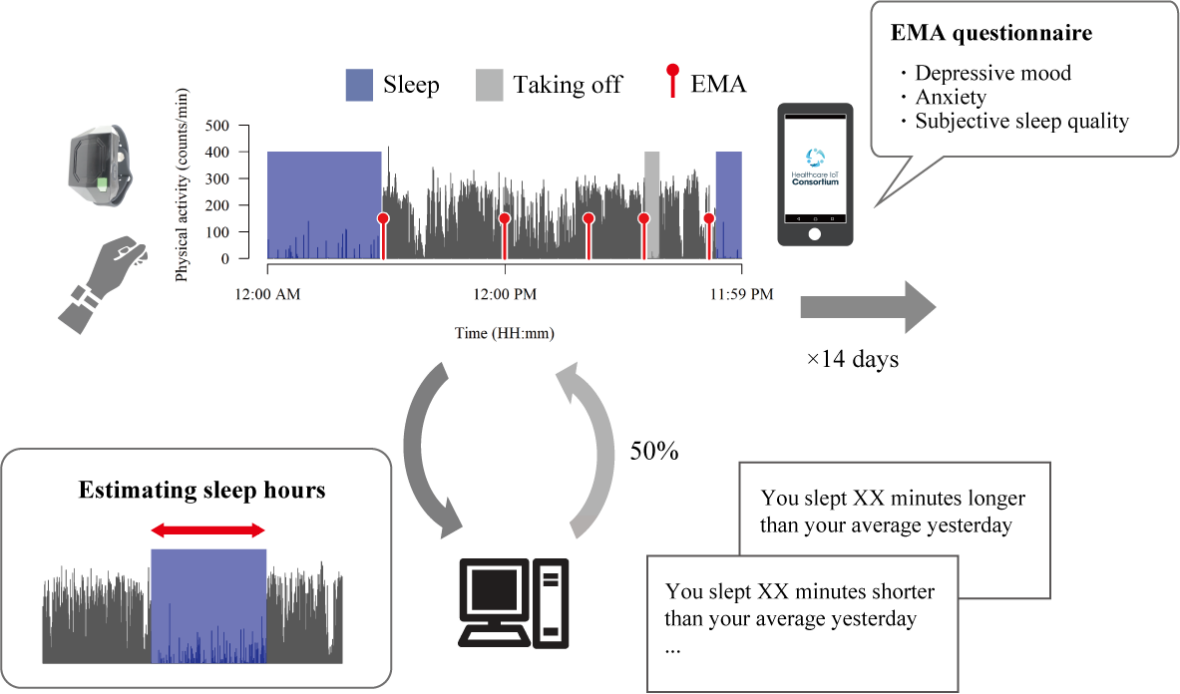
Overview of study 2. As in study 1, participants were instructed to collect sleep data and momentary symptoms using digital devices. In study 2, sleep feedback messages were generated based on their sleep data and provided at random with a 50% chance for each participant over the study period. This message was expected to act as negative feedback when sleep hours were relatively long and as an alert when sleep hours were relatively short. We could examine the within-individual proximal effect of the intervention by comparing these data with and without the feedback. "Taking off" refers to the period that the individual took off the device. EMA: ecological momentary assessment.

We invited 100 workers who had completed study 1, of whom 83 (83%) agreed to participate in study 2 (Figure S2 in Multimedia Appendix 5). Among them, 2 (2%) workers withdrew before writing the consent form because of unexpected schedules. Finally, the sleep JITAI trial (Figure 5) included 81 workers. We excluded all data from 14 (17%) participants from the subsequent analysis because 9 (64%) did not complete the follow-up questionnaire and 5 (36%) had failed to measure sleep behaviors. Table S4 in Multimedia Appendix 6 presents the demographic characteristics of participants whose data were used in the following analyses (n=67; group A: n=23, 34%; group B: n=44, 66%). The 2-tailed Welch *t* test and chi-squared test revealed no substantial differences in demographic characteristics between the groups (Multimedia Appendix 6).

#### Within-Individual Effect of Feedback Messages

We examined the within-individual effect of feedback messages on subsequent sleep hours using a linear mixed-effect model (494 nights in total; Table 1). The model revealed that providing a feedback message significantly prolonged subsequent sleep hours by 40 minutes compared with not providing feedback messages on the first day of the study (*P*=.01). However, a significant interaction was observed between feedback and day (*P*=.04). Thus, the effect of feedback messages decreased by 4 minutes per day over the study period. In addition, subgroup analysis (Table 2) revealed that the effect of the feedback message was only significant in group B (an increase of 59 minutes; *P*=.007), suggesting the potential heterogeneity of effectiveness between the groups. Furthermore, when stratified by cases in which the individual slept longer or shorter than the average in study 1, sleep hours were significantly prolonged after receiving the feedback message, indicating that the previous sleep was shorter than the average (an increase of 1 hour 8 minutes; *P*=.01; Table S5 in Multimedia Appendix 6).

**Table 1:** Within-individual proximal effect of the intervention on subsequent sleep hour.

	Linear mixed-effect model	
	β coefficient (SE; 95% CI)	*P* value
Intercept (no feedback)	6 h 47 min (13 min; 6 h 22 min to 7 h 13 min)	<.001
Age	−2 (1; −4 to −1) min	.001
Sex	35 min (24 min; −11 min to 1 h 20 min)	.15
Sleep hours	2 (3; −3 to 8) min	.46
Feedback	40 min (16 min; 9 min to 1 h 11 min)	.01
Day	2 (1; 0 to 5) min	.09
Feedback×day	−4 (2; −9 to 0 min)	.04

**Table 2:** Subgroup analysis of the within-individual proximal effect.

	Group A		Group B	
	β coefficient (SE; 95% CI)	*P* value	β coefficient (SE; 95% CI)	*P* value
Intercept (no feedback)	7 h 20 min (17 min; 6 h 41 min to 7 h 44 min)	<.001	6 h 32 min (18 min; 6 h 2 min to 7 h 11 min)	<.001
Age	−3 (1; −5 to −1) min	.005	−3 (1; −5 to −1) min	.02
Sex	8 (22; −33 to 49) min	.72	1 h 4 min (46 min; −25 min to 2 h 33 min)	.17
Sleep hours	−3 (5; −12 to 7) min	.60	3 (4; −3 to 10) min	.35
Feedback	−1 (21; −41 to 39) min	.96	59 min (22 min; 17 min to 1 h 42 min)	.007
Day	1 (2; −2 to 5) min	.44	3 (2; −1 to 7) min	.21
Feedback×day	−2 (3; −7 to 4) min	.57	−6 (3; −11 to 0) min	.07

#### Time-Variant Cascading Effect of Feedback Messages on Momentary Symptoms

**Figure 6:**
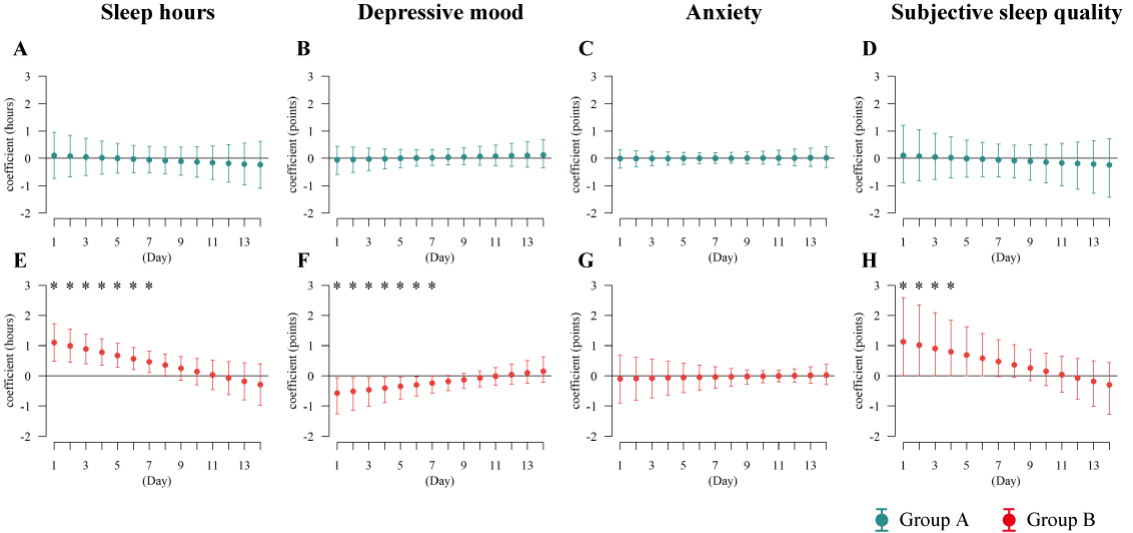
Time-variant effect of the feedback message on subsequent sleep hours and the cascading effect on depressive mood, anxiety, and subjective sleep quality the next morning among group A (A, B, C, and D, respectively) and group B (E, F, G, and H, respectively). The points represent the expected a posteriori of the coefficient, and the error bars indicate the corresponding 95% credible intervals. The asterisk (*) indicates that the 95% credible interval of the estimate did not include the null value.

After performing the Markov chain Monte Carlo sampling, we confirmed that the Gelman-Rubin statistic values for all parameters were <1.10, indicating successful convergence of the posterior distributions of the parameters (Table S6 in Multimedia Appendix 6). On the basis of their point estimates (expected a posteriori) and 95% credible intervals, the model suggested that providing feedback messages substantially prolonged subsequent sleep hours during the first 7 days (Figure 6). Furthermore, it was shown that providing feedback improved depressive mood and subjective sleep quality in the following wake-up time during the first 7 and 4 days, respectively. As sleep feedback was randomly provided, it should be noted that participants did not receive it daily. Table S6 in Multimedia Appendix 6 shows all point estimates and corresponding 95% credible intervals.

#### Comparing Sleep Statistics Between Studies 1 and 2

**Figure 7:**
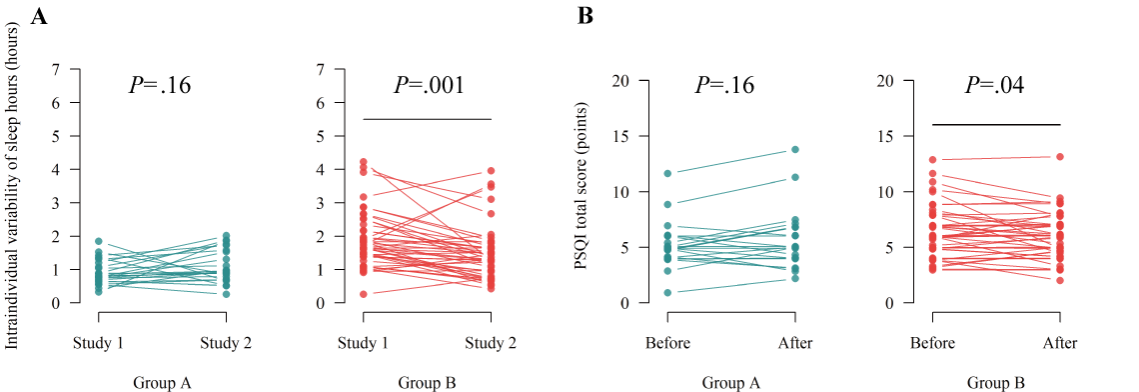
Distal effects on intraindividual variability of sleep hours and the Pittsburgh Sleep Quality Index (PSQI) total score. (A) Changes in the intraindividual variability of sleep hours between studies 1 and 2, (B) changes in the PSQI total score before and after the trial.

Mixed-effect ANOVA models were performed to examine the distal effect of the trial on their sleep statistics (Tables S7 and S8 in Multimedia Appendix 6). The models revealed a significant interaction effect between them for IIV^(SH)^ (*F*_1,65_=10.06; *P*=.002) and IIV^(SE)^ (*F*_1,65_=6.86; *P*=.01). Pairwise comparison tests with Tukey correction demonstrated that IIV^(SH)^ significantly decreased only in group B (Figure 7A; *P*=.001), while significant differences in IIV^(SE)^ were not observed in both groups (group A, *P*=.06; group B, *P*=.07). In addition, the models showed a significant main effect of the study on IIM^(SH)^ (*F*_1,65_=6.07; *P*=.02), and pairwise comparison tests indicated that IIM^(SH)^ was significantly shortened during the trial (study 2) compared with study 1 in the overall sample (*P*=.03). Furthermore, a significant main effect in the groups was observed in IIM^(MID)^ (*F*_1,65_=4.33; *P*=.04), IIM^(SE)^ (*F*_1,65_=15.31; *P*<.001), IIV^(SH)^ (*F*_1,65_=17.02; *P*<.001), IIV^(MID)^ (*F*_1,65_=6.83; *P*=.01), and IIV^(SE)^ (*F*_1,65_=30.53; *P*<.001), although this was evident from study 1 findings.

#### Pretest-Posttest Comparison of PSQI and PANAS Scores

The PSQI total and PANAS scores (positive and negative affect) were assessed before and after the trial, and Table S9 in Multimedia Appendix 6 shows the scores. The mixed-effect ANOVA models (Table S10 in Multimedia Appendix 6) showed a significant main effect of time on PANAS positive (*F*_1,65_=4.60; *P*=.04) and negative affect (*F*_1,65_=4.11; *P*=.046) and a significant interaction between time and group in the PSQI total score (*F*_1,65_=5.60; *P*=.02). The pairwise comparison test revealed that the PSQI total score significantly improved in group B (Figure 7B; *P*=.04), while no significant differences were observed in the PANAS scores (positive affect, *P*=.14; negative affect, *P*=.08).

## Discussion

### Overview

This study aimed to identify a subpopulation with sleep problems in their daily lives using real-world objective sleep data (study 1) and to test whether the sleep feedback messages proximally and distally improved habitual sleep behaviors (study 2). In addition to sleep behaviors, the cascading effect on psychological wellness was examined using EMA recordings, considering the close link between sleep problems and poor psychological wellness [9,10]. Appropriate sleep patterns are essential for promoting the quality of life of workers; several studies have reported an association between sleep problems and adverse occupational phenomena, such as presenteeism [11-13], burnout syndrome [60], low back pain [60,61], and errors at work [62]. Furthermore, a report by the RAND Corporation estimated that sleep problems cost up to US $680 billion annually in 5 Organisation for Economic Co-operation and Development countries (Canada, Germany, Japan, the United States, and the United Kingdom) [63]. The economic loss in Japan was estimated to be approximately US $148 billion annually (equivalent to 2.92% of its GDP), the highest among the 5 countries. Sleep JITAI may solve these occupational and economic problems and disease onset [1-8] by encouraging daily self-management of habitual sleep behaviors.

### Principal Findings: Study 1

In study 1, a hierarchical clustering algorithm was adapted to analyze multidimensional sleep data and identify a subpopulation characterized by larger IIV in sleep data (sleep instability). Recently, the stability of habitual sleep behavior has been considered an additional factor in preventing future health problems [64,65] along with IIMs. Previous studies have indicated that the instability of sleep hours is associated with psychological wellness (including depressive symptoms, anxiety, subjective sleep quality, and psychological well-being) [65-67]. The linear mixed-effect models of this study showed that unstable sleep hours were associated with worse depressive mood, anxiety, and subjective sleep quality in daily life. This result is consistent with the recent findings of the studies that used the EMA method [66], augmenting real-world evidence for this association. Hence, group B, characterized by unstable sleep behaviors, can be regarded as a vulnerable subpopulation that needs sleep management. Along with the instability, individuals in group B showed a delayed sleep-wake schedule (later sleep timing and evening chronotype), larger social jetlag, and lower sleep efficiency, corroborating the necessity for the sleep JITAI for this subpopulation. Further studies are required to elucidate the role of individual circadian rhythms in habitual sleep behavior in real-world settings; however, it has been speculated that their unstable sleep-wake schedule and circadian misalignment caused fragmented sleep during the night, as supported by findings from experimental studies [68,69].

### Principal Findings: Study 2

In study 2, by using MRT, we first examined whether the participants changed their habitual sleep behaviors in response to the sleep feedback. The linear mixed-effect model revealed that sleep hours were prolonged after receiving objective push-type sleep feedback, particularly in group B. On the basis of the control theory [33], the individuals are postulated to change their health behavior to reduce the discrepancy against the set point value. In other words, they might have rather reduced sleep hours after being informed that they slept longer than their set point values (ie, the IIM^(SH)^ in study 1). However, the participants in group B changed sleep hours only after receiving the alerting feedback, indicating that they slept shorter than their set point value. Thus, they might have interpreted the messages indicating the relatively long sleep hours as encouraging messages and adopted strategies maintaining their sleep behaviors. In future, it is necessary to investigate how workers change their sleep hours, when altered set point values are used in feedback messages (eg, the recommended sleep hours of the National Sleep Foundation: 7-9 hours) [16]. Moreover, throughout the analyses, we found no evidence suggesting the effectiveness of the sleep feedback for those in group A. The reduced necessity for sleep intervention might result in the null effect because the participants in group A had relatively healthy sleep habits in study 1. Previous MRT studies also suggested that mHealth interventions were more effective, especially when the individual showed adverse health behaviors [70]. Thus, interventions are likely most effective when just in time. In this study, we reproduced similar findings at the interindividual level. In other words, the findings of this study demonstrate the importance of identifying vulnerable individuals before the trial, as in study 1.

We also discovered that the effect of feedback weakened over time, and the hierarchical Bayesian model predicted that the effect was no longer considerable after 7 days. This attenuation has also been reported in prior MRT studies and is attributed to the habituation of the trial [28]. Personalizing intervention options may reduce habituation risk and improve overall efficacy [28,71]. Therefore, a method to infer the intraindividual behavioral factors leading to better sleep should be developed, and an algorithm to automatically optimize the intervention options during the trial period should be implemented. For example, some studies have proposed adapting reinforcement learning algorithms in mHealth trials to sophisticate and personalize intervention policies over the trial period [72,73]. Incorporating artificial intelligence into mHealth (artificial intelligence–powered mHealth) [74] is a possible future direction for sustainable health management as a part of daily life. The hierarchical Bayesian model also predicted that providing feedback improved depressive mood and subjective sleep quality through changes in sleep hours, at least during the first 4 days. These findings provide novel insights into the immediate psychological response to sleep improvement and encourage the statement that improving habitual sleep behavior contributes to psychological wellness. However, the model quantified the indirect effects of the objective sleep feedback on the momentary symptoms of the next morning and implicitly assumed that providing the sleep feedback does not affect these symptoms independently of changes in sleep hours. Therefore, it is noted that the model should not be used to examine the effects of intervention options, such as relaxation and meditation, that may also directly affect psychological wellness.

Corresponding to the findings in the analyses of the proximal effect, whole effect analyses showed stabilized sleep hours and improved PSQI total scores after study 2, especially in group B. Thus, in this study, we demonstrated the distal effects of sleep JITAI on sleep stability and subjective sleep quality. Distal outcomes are usually achieved through the accumulation of the proximal effect and are the goals of mHealth trials [21,22,26]. However, despite their importance, previous MRT studies did not investigate distal outcomes [28,70]. Future MRTs should incorporate protocols to assess distal outcomes, such as pretest-posttest comparison, to provide a comprehensive understanding of the effectiveness of the MRT. Contrary to expectations, study 2 had decreased IIM^(SH)^ compared with study 1. As this effect was globally observed across the groups, it may be irrelevant to the interventions and could be caused by the influence of social factors, such as changes in work schedules or remote work frequency due to the spread of the COVID-19 pandemic. Future studies should measure data representing lifestyle to analyze the influence of social schedules on sleep. The IIV^(SE)^ showed an interaction effect in the ANOVA model, suggesting that the response pattern differed by group. However, pairwise comparison tests did not show substantial changes for each group. Similarly, the main effects of time on PANAS scores (positive and negative affect) were significant, while post hoc tests did not detect substantial changes. Therefore, we must be conservative when demonstrating the distal effect of the intervention on the stability of sleep efficiency and emotional well-being, providing opportunities for future mHealth trials to clarify these points.

### Strengths and Future Directions

To our knowledge, a previous MRT study focused on sleep hours, physical activity, and mood among medical interns, who were assumed to be affected by a stressful environment [70]. Their 6-month study examined the effects of the intervention and these moderators, suggesting that an individual’s current health state influenced receptivity to interventions. However, in this study, we performed MRT to generate real-world evidence for the effectiveness of the intervention options rather than exploring the factors to consider when constructing a specific JITAI. On the basis of real-world sleep data, we identified individuals in a vulnerable sleep state and specified their sleep problems (sleep instability) before the trial. Furthermore, we used IIM^(SH)^ measured in study 1 to personalize the set point value of the habitual sleep hours and elaborated feedback messages to stabilize their habitual sleep hours. Through these procedures, we made the mHealth intervention just in time and adaptive.

The intervention effects may diminish over time; however, our results indicate that providing push-type sleep feedback can modify sleep instability in the short term. Furthermore, it was suggested that interventions are unnecessary after the successful stabilization of sleep behavior and until sleep instability recurs. As a possible future direction, we can update this sleep JITAI to be more suitable for daily life by continuously tracking individuals’ sleep stability and providing treatments only when their sleep becomes unstable. Such a timely intervention strategy may be widely applicable to address various health behaviors in daily life. Moreover, an integrative health care information system, such as the HIT system, that enables the multifaceted and real-time monitoring of health-related data may be a technical basis for implementing JITAI.

### Limitations

This study had several limitations. First, considering the scalability of mHealth technologies, the sample size of this study was not large. In fact, a previous mHealth sleep intervention trial had included >1000 poor sleepers (patients with insomnia) [32,75]. However, in this study, the data used for the analyses of the proximal effect of the MRT were repeatedly measured by digital devices. These augmented data could contribute to enhancing the statistical power. In fact, ex-post power analysis (Multimedia Appendix 3) showed that the results of the proximal effect among group B had a 77.2% of observed statistical power, which is close to the typical 80% threshold. Second, the trial period was 2 weeks, which is relatively shorter than that of previous mHealth trials (average duration of 6-7 weeks) [32]. How long the participants would continue to use the device if the trial lasted longer is unknown. Consumer-grade wearable trackers are often abandoned after a few months [76], and daily hassles in data collection and unhelpful suggestions are frequently mentioned reasons [76,77]. We implemented push-type sleep feedback in this trial to minimize the burden of accessing sleep data; however, personalizing the intervention options can also address this issue. Finally, this study included workers who were healthy enough to engage in a typical work schedule in daily life. Therefore, the applicability of this intervention in clinical trials for sleep disorders (eg, insomnia and circadian rhythm sleep disorders) remains unclear. Further studies are warranted to determine whether a similar effect is observed in the clinical population. In addition, whether additive or synergistic effects are observed when incorporating JITAI methodologies into traditional pharmacological or psycho-behavioral sleep interventions should be investigated.

### Conclusions

In conclusion, we provide real-world evidence that objective push-type sleep feedback proximally prolongs sleep hours and distally improves sleep stability and subjective sleep quality. Timely interventions powered by real-time health monitoring and personalized feedback may offer opportunities for individuals in vulnerable health states to manage their health behaviors in daily life. Furthermore, incorporating MRT into a traditional trial design can be helpful to comprehensively understand the effectiveness of the interventions and increase the sophistication of the trial design.

## Appendix

Multimedia Appendix 1 Screenshots of the Health care Internet of Things app.

Multimedia Appendix 2 Validity of the Sciencenet activity monitor.

Multimedia Appendix 3 Explanation of hierarchical Bayesian model.

Multimedia Appendix 4 Flow diagram of studies 1 and 2.

Multimedia Appendix 5 Supplementary data.

Multimedia Appendix 6 The ex-post power analysis.

## Abbreviations

BDI-II: Beck Depression Inventory second edition
BLE: Bluetooth Low Energy
EMA: ecological momentary assessment
HIT: health care Internet of Things
IIM: intraindividual mean
IIV: intraindividual variability
JITAI: just-in-time adaptive intervention
MCTQ: Munich Chronotype Questionnaire
MEQ: Morningness-Eveningness Questionnaire
mHealth: mobile health
MRT: microrandomized trial
MSFsc: sleep-corrected midpoint of sleep on work-free days
PANAS: Positive and Negative Affect Schedule
PSQI: Pittsburgh Sleep Quality Index
STAI: State-Trait Anxiety Inventory-JZY

## Data Availability

The data sets generated during and analyzed during this study are not publicly available because the participants of this study did not agree to their data being shared publicly, but are available from the corresponding author on reasonable request.
