# Supplementary material for: Just-in-Time Adaptive Intervention for Stabilizing Sleep Hours of Japanese Workers: Microrandomized Trial
Source: J Med Internet Res. 2024 Jun 11;26:e49669. doi: 10.2196/49669 (PMC11200036; doi:10.2196/49669)
Supplement: Multimedia Appendix 1 [file jmir_v26i1e49669_app1.doc]

Multimedia Appendix 1: Screenshots of the HIT app


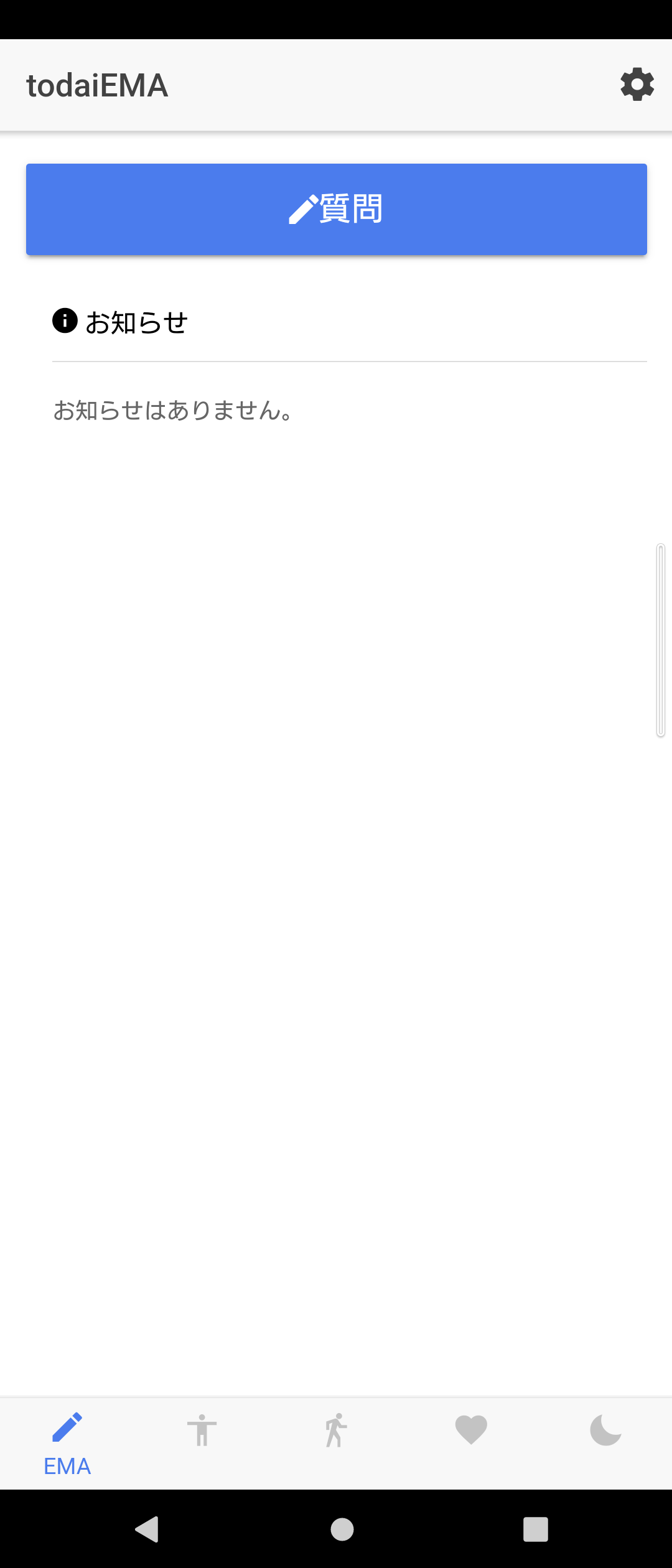


Figure. S1: Home screen of the HIT app


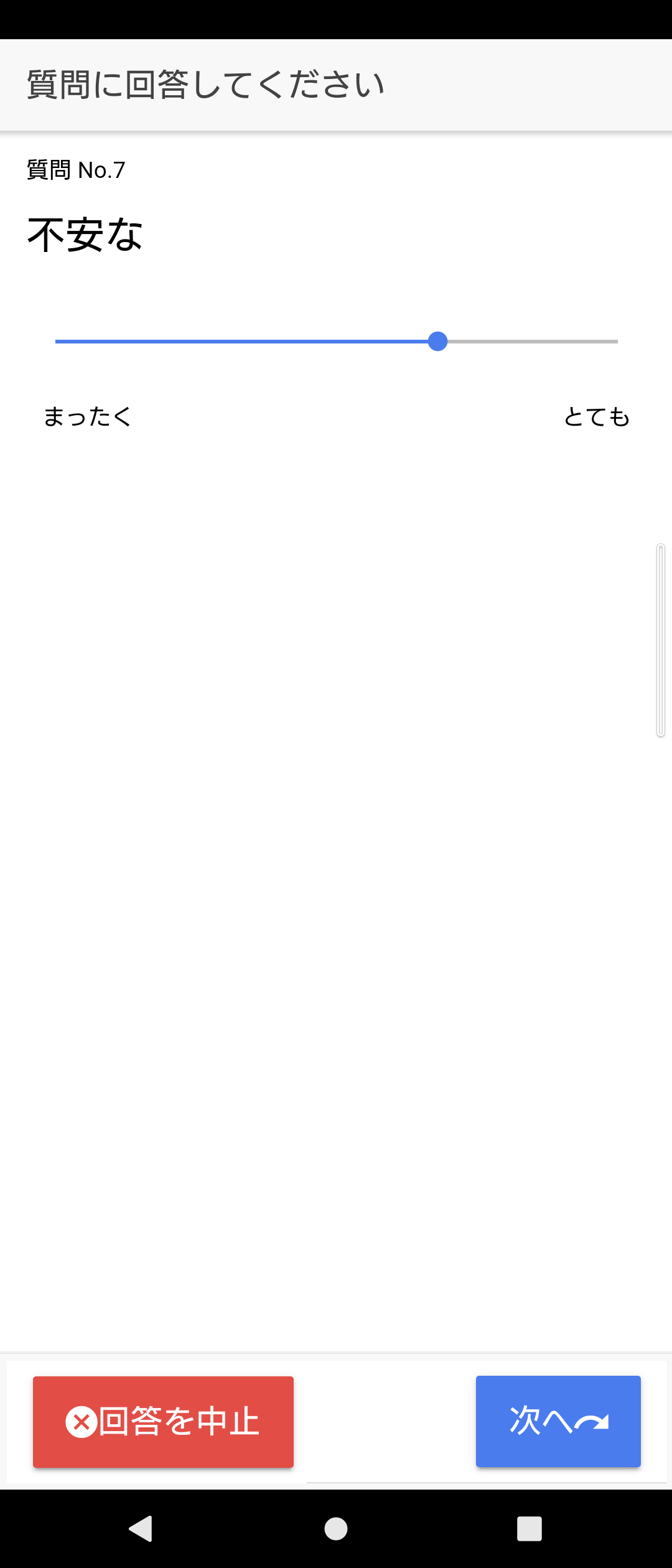


Figure. S2: EMA screen of the HIT app

By tapping the [質問] icon on the home screen, the users can answer the EMA questionnaires. The EMA measurements were rated using a visual analog scale from 0 to 100 displayed on the screen.


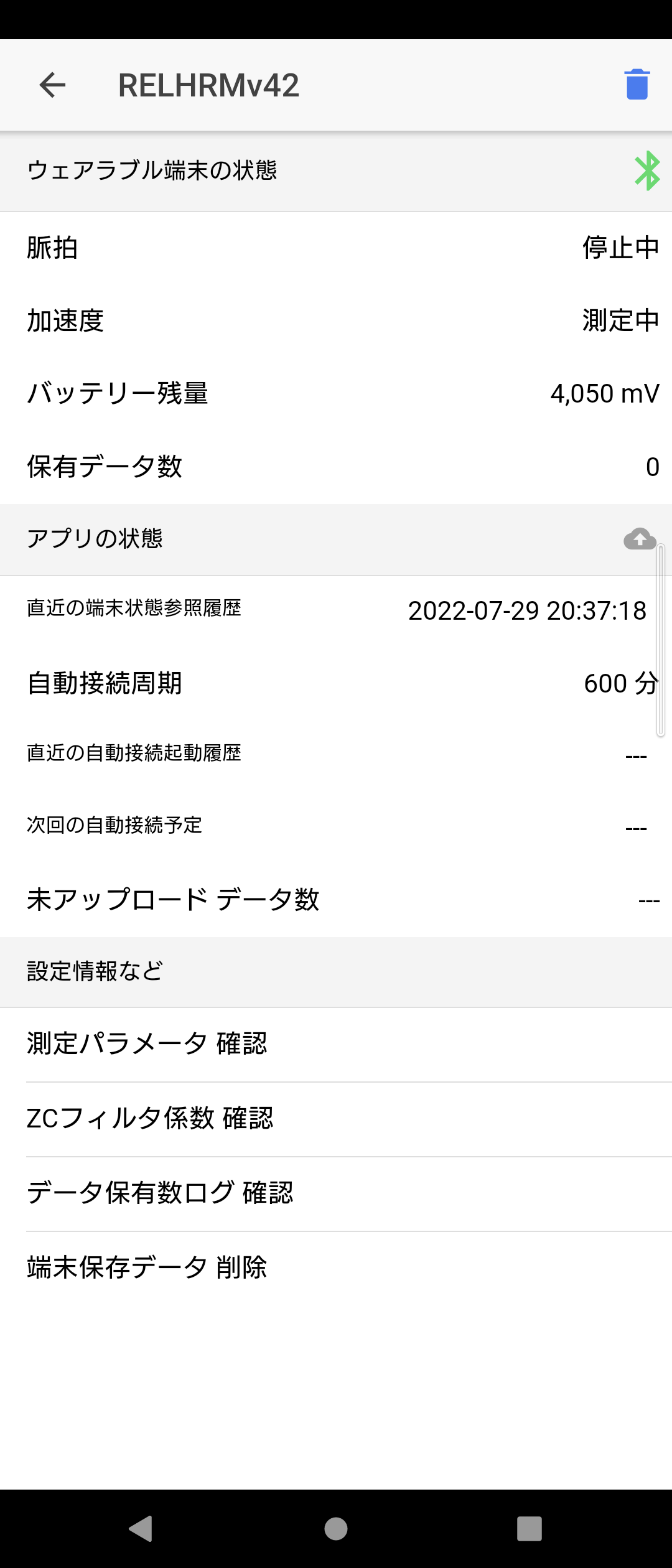


Figure. S3: Pairing screen of the HIT app

The users can check the measurement status of the wearable activity monitor (stopping or recording) and battery status.


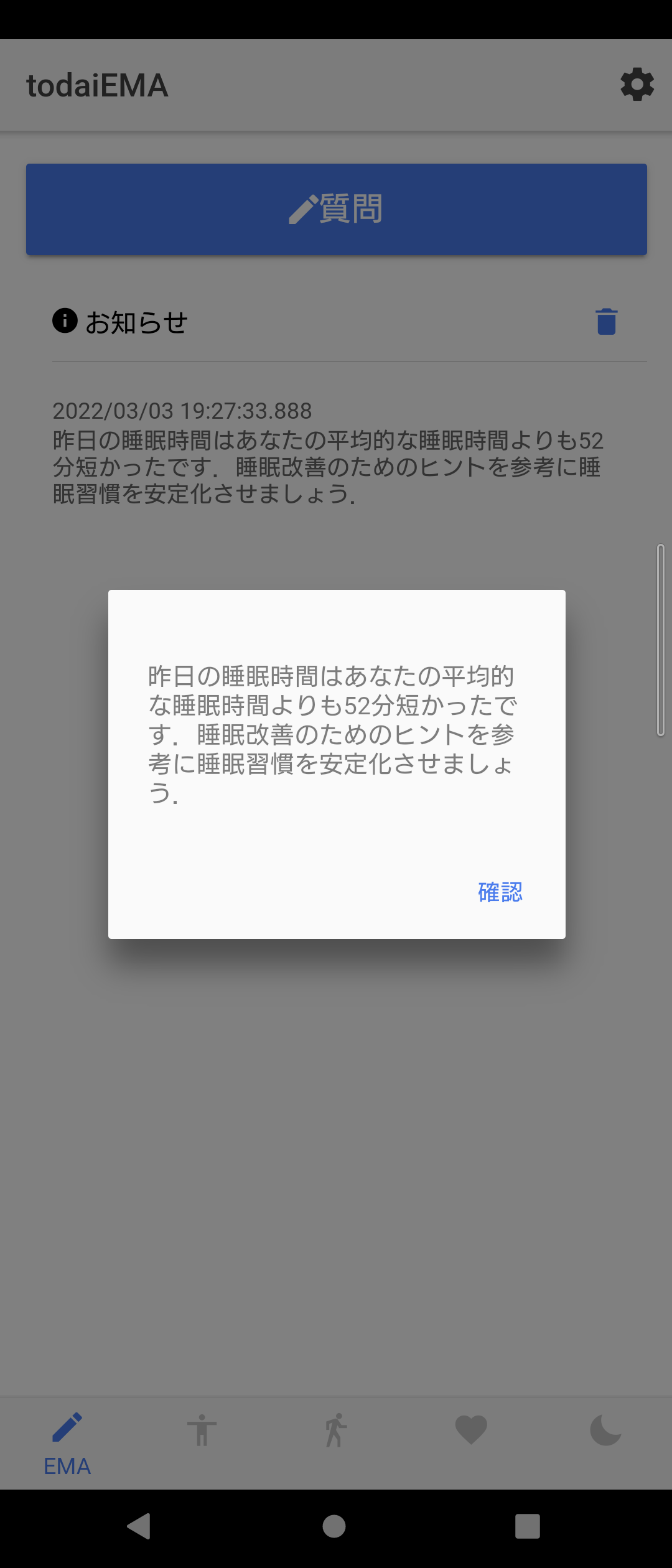


Figure. S4: An example of personalized sleep feedback.

During the survey period, the personalized sleep feedback was generated based on objective measurements and popped up on the screen at 12 PM. This figure shows an example of the message; You slept 52 minutes shorter than your average yesterday. Stabilize your sleep habits with reference to sleep hygiene guidelines.”
