# Supplementary material for: Just-in-Time Adaptive Intervention for Stabilizing Sleep Hours of Japanese Workers: Microrandomized Trial
Source: J Med Internet Res. 2024 Jun 11;26:e49669. doi: 10.2196/49669 (PMC11200036; doi:10.2196/49669)
Supplement: Multimedia Appendix 2 [file jmir_v26i1e49669_app2.doc]

Multimedia Appendix 2: Validity of Sciencenet activity monitor

To obtain the zero-crossing counts (ZC) data of the Sciencenet device, we first filtered the recorded body acceleration data with a 6th order Butterworth filter from 2 to 3 Hz. Thereafter, we counted the number of times the signal level crossed 0.01 G within an epoch for each axis. Finally, the largest value among the axes was used as the ZC data for the epoch.


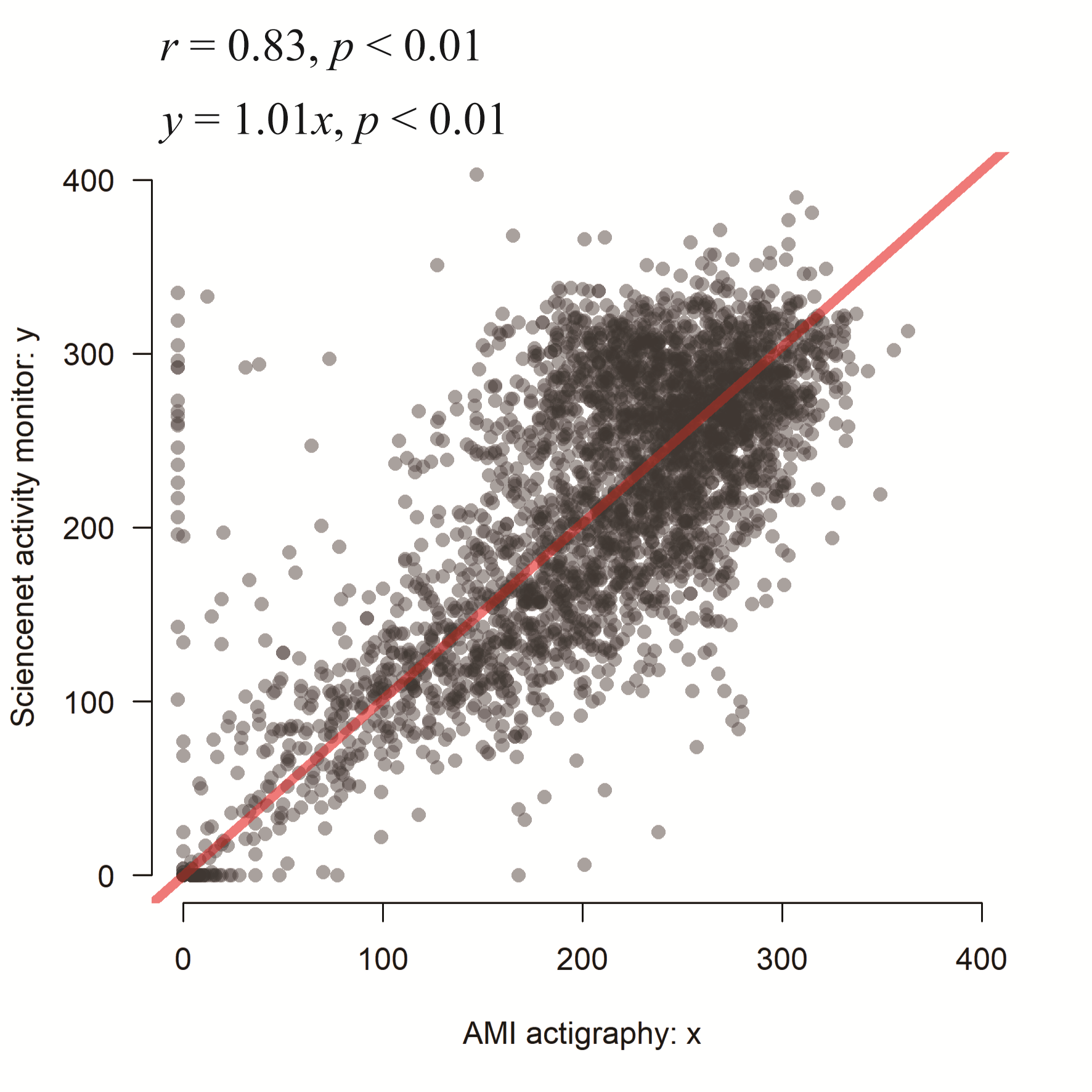


Figure. S1: Scatter plot of ZC data.

The horizontal and vertical axes represent the ZC data of the AMI actigraphy* and Sciencenet activity monitor, respectively. Data were recorded simultaneously from a specific participant for three days. We examined the validity of our in-house activity monitor using ZC data when the participant was awake. Correlation analysis showed a strong correlation between them (*r* = 0.83, *p* < 0.01), and the slope of the linear regression model was 1.01 (p < 0.01) when the intercept was assumed to be zero. These results indicate that the ZC data of our in-house device were comparable with those of the AMI actigraphy.

*The AMI actigraphy (Ambulatory Monitors Inc., Ardsley, NY, USA) has been approved by the Food and Drug Administration in the United States and widely used in clinical settings.

| Accuracy: 0.929  Sensitivity: 0.896  Specificity: 0.995 | | Sciencenet | |
| --- | --- | --- | --- |
|  |  | Wake | Sleep |
| AW2 | Wake | 3362 | 392 |
|  | Sleep | 9 | 1907 |

Table S1: Confusion matrix (in epochs) for sleep-wake identification.

We compared the performance of epoch-by-epoch sleep-wake identification between the AMI actigraphy and Sciencenet devices. The Cole-Kripke algorithm with Webster’s rescoring rules was adopted for the physical activity data of the Sciencenet device to identify whether the 1-minute epoch was sleep or wake. Thereafter, we used the same algorithm on the Action-W Version 2 software for analyzing the AMI actigraphy data. When comparing these outputs, the accuracy, sensitivity, and specificity were 0.929, 0.896, and 0.995, respectively.


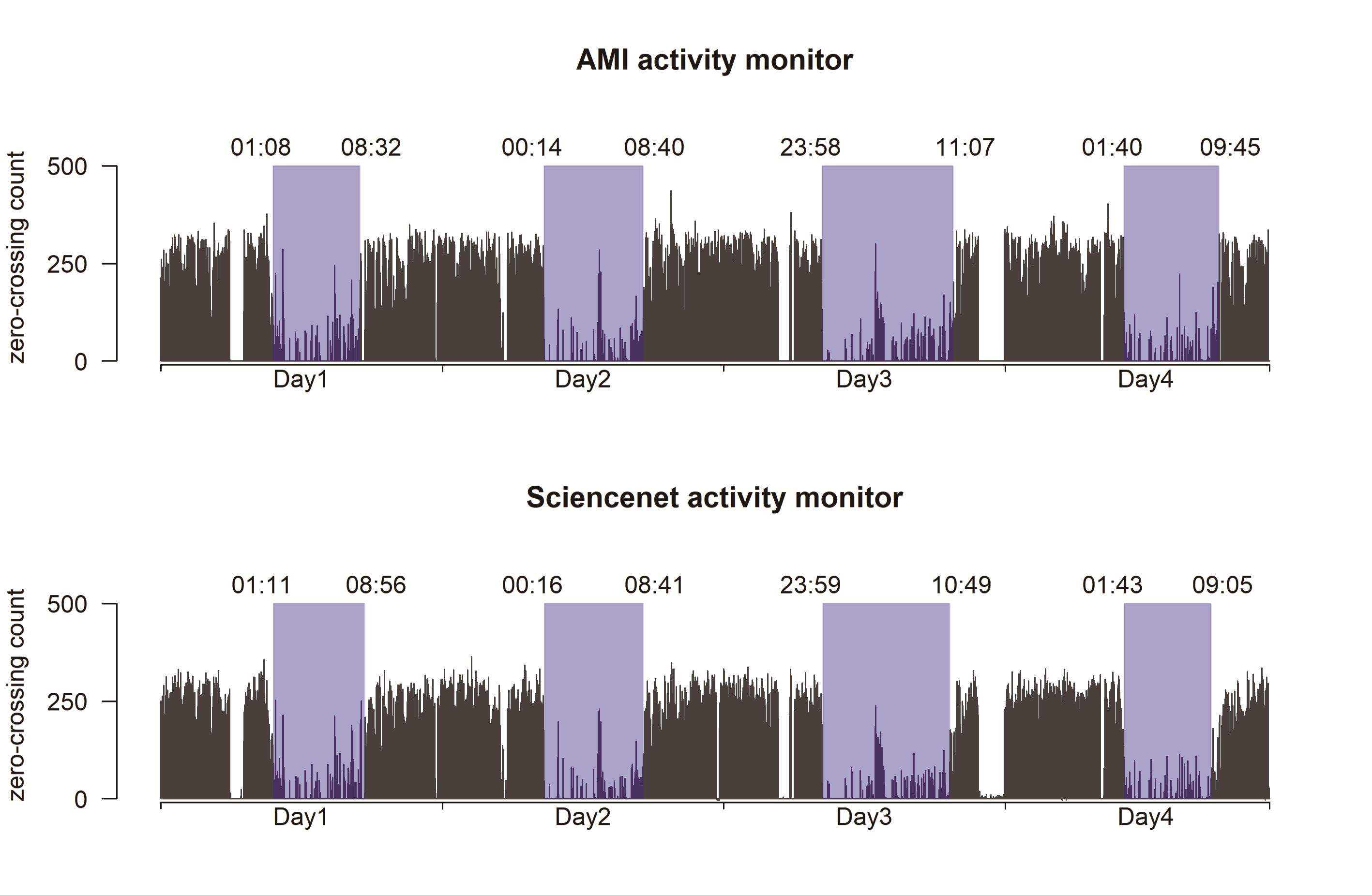


Figure. S2: Representative data of estimated sleep intervals using the AMI and Sciencenet activity monitors.

The blue areas in the top and bottom panels indicate the estimated sleep intervals with Action-W Version 2 software and the dedicated Sciencenet algorithm, respectively. The figure indicates that the Sciencenet device and algorithm are analogous in performance to research-grade actigraphy.
