# Supplementary material for: Just-in-Time Adaptive Intervention for Stabilizing Sleep Hours of Japanese Workers: Microrandomized Trial
Source: J Med Internet Res. 2024 Jun 11;26:e49669. doi: 10.2196/49669 (PMC11200036; doi:10.2196/49669)
Supplement: Multimedia Appendix 3 [file jmir_v26i1e49669_app3.docx]

**Multimedia Appendix 3: Explanation of hierarchical Bayesian model**

Using hierarchical Bayesian modelling, we performed counterfactual simulations to examine 1) the number of days the feedback message was effective for subsequent sleep, and 2) whether the changes in sleep hours were linked to improvements in psychological wellness (depressive mood, anxiety, and subjective sleep quality). In particular, we estimated the cascading effect of feedback on momentary symptoms in the next morning through changed sleep hours as shown below:

$$S_{\left( i+1 \right)j}=\alpha_{j}^{\left( 0 \right)'}+\alpha^{\left( 1 \right)}\left( S_{ij}-\mathrm{IIM}_{j}^{(SH)} \right)+\left( \alpha^{\left( 2 \right)'}+\alpha^{\left( 3 \right)'}D_{ij} \right)I_{ij}+\alpha^{\left( 4 \right)}D_{ij}+e_{ij}^{(\alpha)}\ldots(1.1)$$

$\alpha_{j}^{\left( 0 \right)'}=\alpha_{j}^{\left( 0 \right)}+\alpha^{\left( 5 \right)}G_{i}\ldots(1.2)$

$\alpha^{\left( 2 \right)'}=\alpha^{\left( 2 \right)}+\alpha^{\left( 6 \right)}G_{i}\ldots(1.3)$

$\alpha^{\left( 3 \right)'}=\alpha^{\left( 3 \right)}+\alpha^{\left( 7 \right)}G_{i}\ldots(1.4)$

$$\alpha_{j}^{\left( 0 \right)}\sim N\left( \alpha^{(0)}, \tau^{\left( \alpha\right)} \right)\ldots(1.5)$$

$e_{ij}^{(\alpha)}\sim N\left( 0,\sigma^{\left( \alpha\right)} \right)\ldots(1.6)$

$${Depressive mood}_{\left( i+1 \right)j}=\beta_{j}^{\left( 0 \right)}+\beta^{\left( 1 \right)}\left( S_{\left( i+1 \right)j}-\mathrm{IIM}_{j}^{(SH)} \right)+e_{ij}^{\left( \beta\right)}\ldots\left( 2.1 \right)$$

$$\beta_{j}^{\left( 0 \right)}\sim N\left( \beta^{(0)}, \tau^{\left( \beta\right)} \right)\ldots(2.2)$$

$$e_{ij}^{(\beta)}\sim N\left( 0,\sigma^{\left( \beta\right)} \right)\ldots(2.3)$$

$${Anxiety}_{\left( i+1 \right)j}=\gamma_{j}^{\left( 0 \right)}+\gamma^{\left( 1 \right)}\left( S_{\left( i+1 \right)j}-\mathrm{IIM}_{j}^{(SH)} \right)+e_{ij}^{\left( \gamma\right)}\ldots\left( 3.1 \right)$$

$$\gamma_{j}^{\left( 0 \right)}\sim N\left( \gamma^{(0)}, \tau^{\left( \gamma\right)} \right)\ldots(3.2)$$

$$e_{ij}^{(\gamma)}\sim N(0, \sigma^{\left( \gamma\right)})\ldots(3.3)$$

$${Sleep quality}_{(i+1)j}=\delta_{j}^{(0)}+\delta^{\left( 1 \right)}\left( S_{(i+1)j}-\mathrm{IIM}_{j}^{(SH)} \right)+e_{ij}^{(\delta)}\ldots(4.1)$$

$$\delta_{j}^{\left( 0 \right)}\sim N\left( \delta^{(0)}, \delta^{\left( \alpha\right)} \right)\ldots(4.2)$$

$$e_{ij}^{(\delta)}\sim N(0, \sigma^{\left( \delta\right)})\ldots(4.3)$$

S_ij_ indicates the sleep hours at the i-th (i = 1, 2, …, 14) recording of the j-th (j = 1, 2, …, 67) individual. Depressive mood_(i+1)j_, anxiety _(i+1)j_, and sleep quality_(i+1)j_ indicate momentary symptoms recorded in the wake-up EMA. IIM^(SH)^_j_ indicates the IIM^(SH)^ of the j-th individual estimated in study 1. I_ij_ indicates whether feedback was provided (I_ij_ = 1) or not (I_ij_ = 0). D_ij_ is the index representing the elapsed day from the beginning of the survey (D_ij_ = 0 denoted that the corresponding data was recorded on the first day of the survey). G_i_ is the index representing the group where the participant was classified (G_i_ = 0, group A; G_i_ = 1, group B). Therefore, this model assumed that the effect of the feedback message differed by the group and changed over the survey period. e^(α)^_ij_, e^(β)^_ij_, e^(γ)^_ij_, and e^(δ)^_ij_ indicate the residuals following normal distributions and σ^(α)^, σ^(β)^, σ^(γ)^, and σ^(δ)^ represent their SDs. α^(0)^_j_, β^(0)^_j_, γ^(0)^_j_, and δ^(0)^_j_ are the random intercepts of the j-th participants and are assumed to be generated from the normal distributions. Thus, α^(0)^, β^(0)^, γ^(0)^, and δ^(0)^ represent their expectations and τ^(α)^, τ^(β)^, τ^(γ)^, and τ^(δ)^ represent their SDs. The other parameters (α, β, γ, and δ) without subscripts indicate the coefficients of the corresponding variables. Specifically, (α^(2)’^ + α^(3)’^ D_ij_) represents the time-variant effect of the feedback message on subsequent sleep hours for each group. We examined how long the feedback substantially improved their sleep by computing their 95% credible intervals. As shown below, by substituting (1.1) into (2.1), (3.1), and (4.1), the cascading effects of feedback on these symptoms were calculated using the products of (α^(2)’^ + α^(3)’^ D_ij_) with β^(1)^, γ^(1)^, and δ^(1)^:

$$Time-variant effect =\left( \alpha^{\left( 2 \right)'}+\alpha^{\left( 3 \right)'}D_{ij} \right)\ldots\left( 5.1 \right)$$

$$\mathrm{Cascading}\mathrm{effect}^{(\beta)}=\beta^{(1)}*\left( \alpha^{\left( 2 \right)'}+\alpha^{\left( 3 \right)'}D_{ij} \right)\ldots\left( 5.2 \right)$$

$$\mathrm{Cascading}\mathrm{effect}^{(\gamma)}=\gamma^{(1)}*\left( \alpha^{\left( 2 \right)'}+\alpha^{\left( 3 \right)'}D_{ij} \right)\ldots\left( 5.3 \right)$$

$$\mathrm{Cascading}\mathrm{effect}^{(\delta)}=\delta^{(1)}*\left( \alpha^{\left( 2 \right)'}+\alpha^{\left( 3 \right)'}D_{ij} \right)\ldots\left( 5.4 \right)$$

Uniform distribution (-∞, +∞) was used as prior distribution for these parameters. These parameters and their 95% credible intervals were computed using the “rstan” package. The number of chains was set to four when performing the Markov Chain Monte Carlo sampling. All iterations and burn-in samples were set to 8,000 and 4,000, respectively. Convergence across the four chains was defined as when the value of the Gelman-Rubin statistic (Rhat) for all parameters was < 1.10. The model was run after controlling for age and sex.
