# Supplementary material for: Just-in-Time Adaptive Intervention for Stabilizing Sleep Hours of Japanese Workers: Microrandomized Trial
Source: J Med Internet Res. 2024 Jun 11;26:e49669. doi: 10.2196/49669 (PMC11200036; doi:10.2196/49669)
Supplement: Multimedia Appendix 4 [file jmir_v26i1e49669_app4.pptx]

## Slide 1
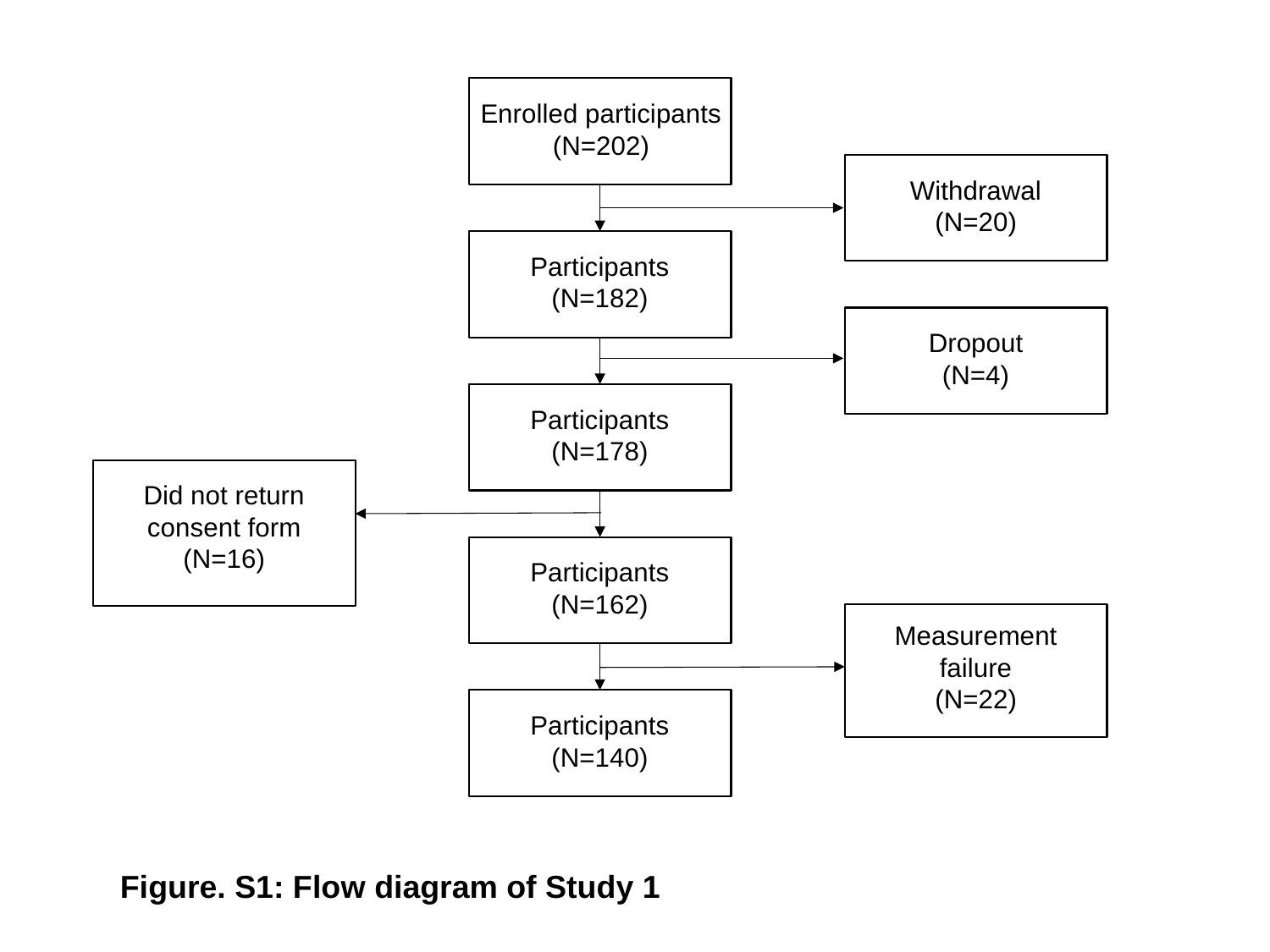

Enrolled participants
(N=202)
Withdrawal
(N=20)
Participants
(N=182)
Dropout
(N=4)
Participants
(N=178)
Did not return consent form
(N=16)
Participants
(N=162)
Measurement failure
(N=22)
Participants
(N=140)
Figure. S1: Flow diagram of Study 1

## Slide 2
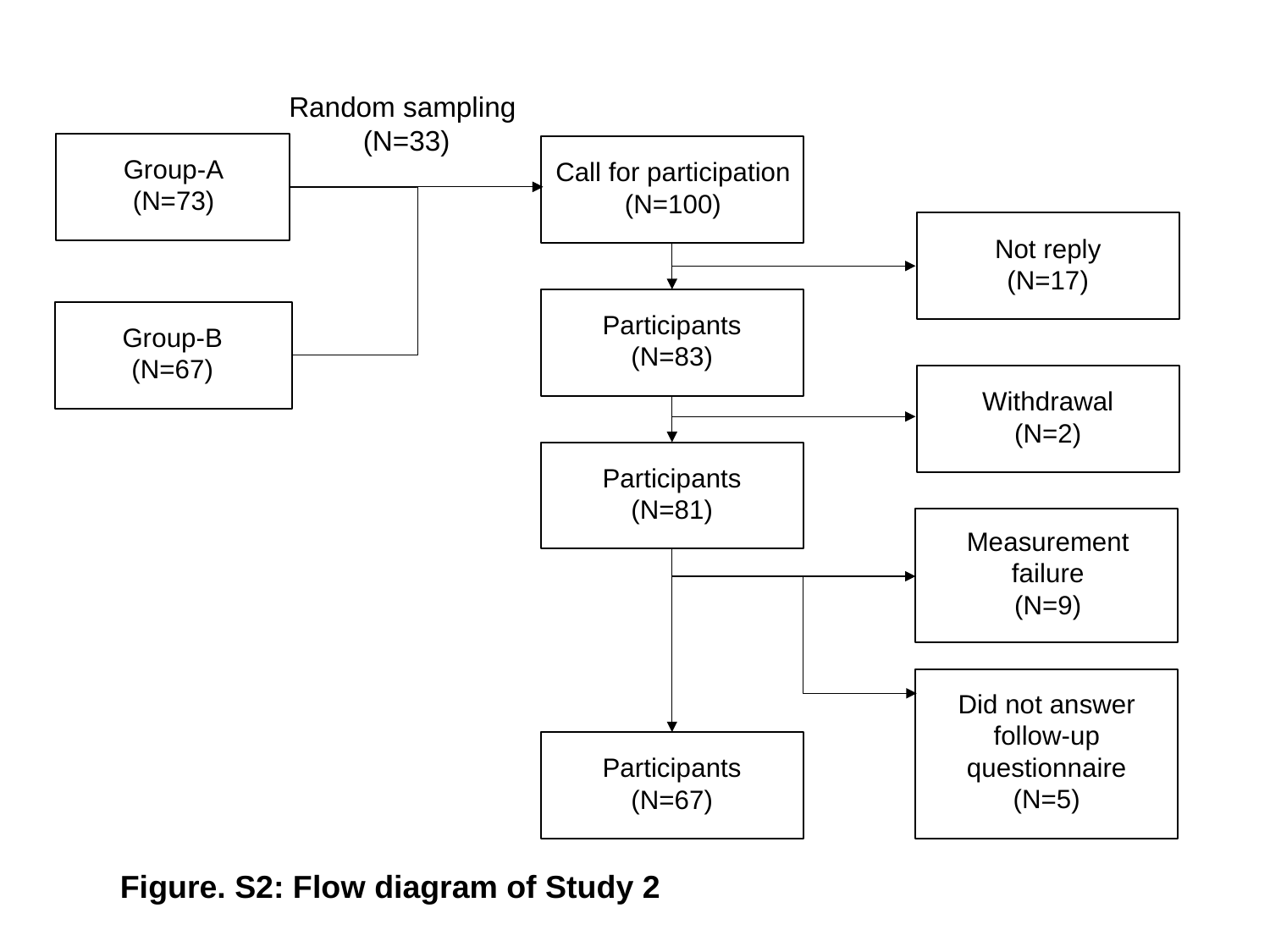

Random sampling
(N=33)
Group-A
(N=73)
Call for participation
(N=100)
Not reply
(N=17)
Participants
(N=83)
Group-B
(N=67)
Withdrawal
(N=2)
Participants
(N=81)
Measurement failure
(N=9)
Did not answer follow-up questionnaire
(N=5)
Participants
(N=67)
Figure. S2: Flow diagram of Study 2
