# Supplementary material for: Just-in-Time Adaptive Intervention for Stabilizing Sleep Hours of Japanese Workers: Microrandomized Trial
Source: J Med Internet Res. 2024 Jun 11;26:e49669. doi: 10.2196/49669 (PMC11200036; doi:10.2196/49669)
Supplement: Multimedia Appendix 6 [file jmir_v26i1e49669_app6.docx]

**Multimedia Appendix 6: The ex-post power analysis**

Conducting power analysis for linear mixed-effect models requires to infer not only effect size of the interested fixed effect but also within and between group (or individual) variance parameters in the model, making it relatively intricate compared to the case of general liner models. The “simr” package in R statistical software can perform power analysis for the interested fixed effect in generalized linear mixed-effect models by using Monte Carlo simulation.

We performed ex-post power analysis by using the “simr” package particularly to calculate observed statistical power of the feedback message and identify the approximate minimum sample size. These simulations can provide an indication of sample size in future studies.

1. **Calculating observed statistical power**

In the manuscript, we performed the linear mixed-effect model to examine the proximal effect of sleep feedback message (see Method section or Table 1 and 2). This model incorporated an indicator of whether feedback was provided or not (variable “feedback”), an index representing the elapsed day from the beginning of the survey (variable “day”), and their interaction effect (variable “feedback × day”). Particularly, coefficient of the “feedback” indicates the effect of the feedback message on the first day of the trial.

In this section, we calculated the observed statistical power for the feedback effect in vulnerable sleepers (group B, n = 44) based on the model corresponding to Table 2 of the manuscript. This is because the result is substantially relevant to our discussion and conclusion.

The simulation was performed with 1,000 Monte Carlo simulations. As shown below, the result indicates that our analysis exhibited a moderate statistical power (mean = 77.20%, 95%CI = [74.47%, 79.77%]), which is close to the typical 80% threshold.

## Power for predictor 'feedback1', (95% confidence interval):
## 77.20% (74.47, 79.77)
##
## Test: t-test with Satterthwaite degrees of freedom (package lmerTest)
## Effect size for feedback1 is 59.
##
## Based on 1000 simulations, (0 warnings, 0 errors)
## alpha = 0.05, nrow = 315
##
## Time elapsed: 0 h 1 m 53 s
##
## nb: result might be an observed power calculation

1. **Identifying the approximate minimum sample size**

In addition to calculating the observed statistical power, we identified the approximate minimum sample size that surpasses the 80% threshold. We generated simulation datasets based on the observed data with the “extend” function and plotted a response curve of statistical power with respect to increments in sample size. We augmented the sample size by 5 for each trial and conducted the simulations with a dataset containing up to 70 individuals, which corresponds to the approximate number of vulnerable sleepers identified in study 1.


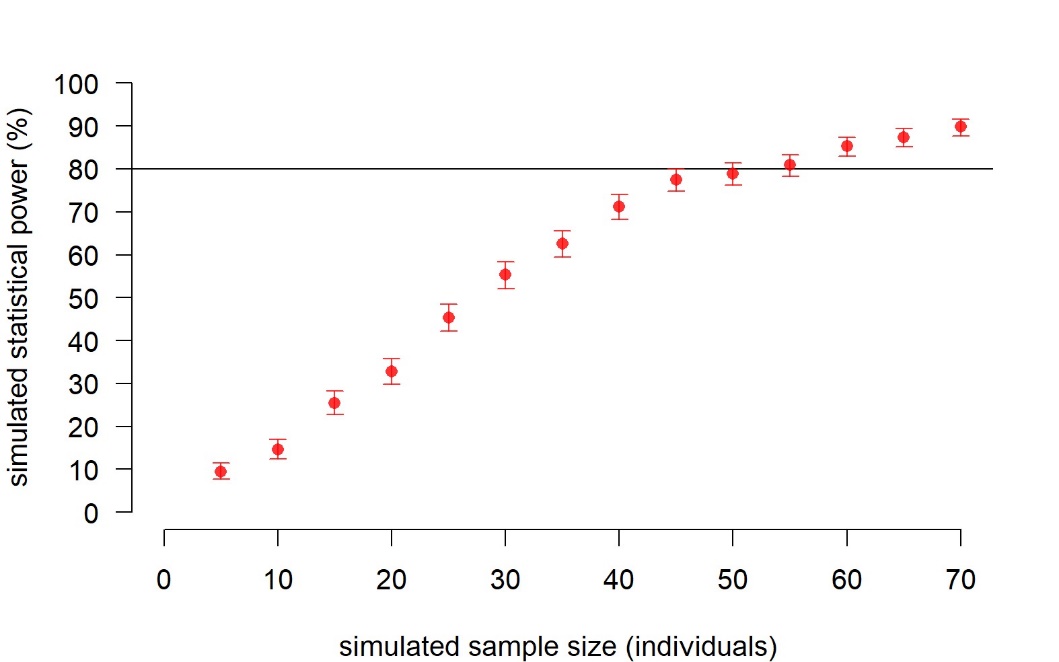


Figure. S1: The simulated response curve of statistical power

The simulations were performed with 1,000 Monte Carlo sampling for each trial.

Based on the simulation results (Figure. S1), the approximate minimum sample size, which exceeds the 80% threshold, was N = 60. At the same time, these results suggest that the total number of vulnerable sleepers identified in study 1 (N = 67) would have been sufficient in terms of statistical power; however, it is noted that we could not include all of them in the sleep JITAI due to the time and budgetary constraints.
